# Environmental surface chemistries and adsorption behaviors of metal cations (Fe^3+^, Fe^2+^, Ca^2+^ and Zn^2+^) on manganese dioxide-modified green biochar†

**DOI:** 10.1039/c9ra03112j

**Published:** 2019-08-02

**Authors:** Panya Maneechakr, Surachai Karnjanakom

**Affiliations:** Department of Chemistry, Faculty of Science, Rangsit University Pathumthani 12000 Thailand panya.m@rsu.ac.th

## Abstract

The facile preparation and modification of low-cost/efficient adsorbents or biochar (CP) derived from the carbonization of palm kernel cake (lignocellulosic residue) has been studied for the selective adsorption of various metal cations, such as Fe^3+^, Fe^2+^, Ca^2+^ and Zn^2+^, from aqueous solution. The CP surface was modified with KMnO_4_ (CPMn) and HNO_3_ (CPHNO_3_) in order to improve the adsorption efficiency. The physicochemical properties of the as-prepared adsorbents were investigated *via* BET, pH_pzc_, FT-IR, Boehm titration, TG-DTG, XRD and SEM-EDS techniques. The surfaces of all adsorbents clearly demonstrated negative charge (pH_pzc_ > pH of the mixture solution), resulting in a high adsorption capacity for each metal cation. Fe^2+^ was found to be more easily adsorbed on modified CP than the other kinds of metal cations. Synergistic effects between the carboxylic groups and MnO_2_ on the surface of CPMn resulted in better performance for metal cation adsorption than was shown by CPHNO_3_. The maximum adsorption capacities for Fe^3+^, Fe^2+^, Ca^2+^ and Zn^2+^ using CPMn, which were obtained from a monolayer adsorption process *via* Langmuir isotherms (*R*^2^ > 0.99), were 70.67, 68.60, 5.06 and 22.38 mg g^−1^, respectively. The adsorption behavior and monolayer-physisorption behavior, *via* a rapid adsorption process as well as single-step intra-particle diffusion, were also verified and supported using Dubinin–Radushkevich, Redlich–Peterson and Toth isotherms, a pseudo-second-order kinetic model and the Weber–Morris model. Moreover, the thermodynamic results indicated that the adsorption process of metal cations onto the CPMn surface was endothermic and spontaneous in nature. This research is expected to provide a green way for the production of low-cost/efficient adsorbents and to help gain an understanding of the adsorption behavior/process for the selective removal of metal ions from wastewater pollution.

## Abstract 2

Manganese dioxide-modified green biochar exhibited excellent capacity for adsorption of Fe^3+^, Fe^2+^, Ca^2+^ and/or Zn^2+^.

## Graphical abstract

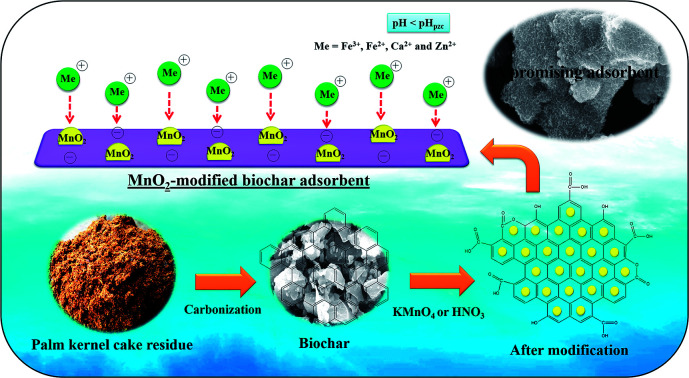

## 1. Introduction

The existence of toxic/heavy metals in water is regarded as a part of the inorganic pollution that has become a worldwide issue, due to their deleterious effects on the environment, including human life and ecological systems.^1,2^ Recently, Fe, Zn and Ca have raised concern and been identified as toxic metals that are generally discovered in ground/surface water, leading to many problems in industrial processes, such as slag formation in boilers and tube failure. In fact, the concentrations of Fe and Zn, as reported by the World Health Organization (WHO), should not exceed over 0.3 and 0.01 mg L^−1^, respectively. The three water treatment or purification steps in industrial processes are: (I) the removal of suspended solids from the system *via* precipitation; (II) the removal of organic substances using bacterial degradation; and (III) the removal of inorganic substances including various metals.^1,4–6^ Several conventional technologies such as coagulation, co-precipitation, filtration membranes and ion-exchange have been mainly identified as good processes for the removal of metal ions from wastewater.^3,7,8^ However, the disadvantages of these technologies include the high costs of equipment, the large amount of expensive chemicals required and the complications of large-scale operations. Adsorption processes have been proven to be an effective and reliable method for the removal of metal ions from wastewater.^9^ Activated carbon is accepted as an excellent adsorbent since it presents a high surface area, large porosity and long-term stability. The development and modification of carbon surfaces results in improved adsorption rates and capacities.^10^ Commercial activated carbon is chiefly used in practical wastewater treatment processes. However, it involves higher production costs than charcoal, for instance, as the pyrolysis process is performed at high temperatures of ∼900–1000 °C under a N_2_ atmosphere using hardwoods as the feedstocks.^11^ Recently, it has been found that the performance of commercial activated carbon is solely limited to the excellent adsorption of non-polar or slightly polar molecules, such as reactive dyes, iodine, and organic compounds (lipids and phenols); however, the adsorption capacities for polar molecules, including cations and anions such as Ca^2+^, Mg^2+^, Cr^3+^, Cd^2+^, Fe^2+^, Fe^3+^, Zn^2+^, AsO_4_^3−^, Cr_2_O_7_^2−^ and MnO_4_^−^, are quite low.^12^ For cation adsorption mechanisms, the existence of oxygen/nitrogen functional groups is required, such as carboxylic, hydroxyl and amine groups with lone pair electrons, which could serve as Lewis bases on the surface of activated carbon for the attraction of cations (Lewis acids) *via* electrostatic forces with the generation of co-ordinate covalent bonds. As mentioned above, it is still necessary to develop and improve the selective functional groups on the surface of activated carbon to improve the adsorption capacity and selectivity for cations. To date, many researchers have been searching for effective waste biomass to use for the production of low-cost and highly efficient activated carbons.

In our previous work, we studied the production of activated carbon from waste lignocellulosic biomass, such as *Terminalia catappa* seeds and *Cerbera odollam* seeds, and the results indicated that these activated carbon sources exhibited better adsorption capacities for Ca^2+^, Fe^2+^, Fe^3+^ and Cr^3+^ than commercial activated carbon. It is also considered that the production costs are much cheaper, compared with commercial activated carbon.^12^ However, due to the existence of too small amounts of these kinds of waste biomass sources, in a further idea we tried to apply charcoal sold in a local market as an adsorbent feedstock. The advantages of charcoal or biochar are: (I) the production costs are low; (II) the production process is not complicated; and (III) villagers can produce by themselves great amounts of this adsorbent. It should be mentioned here that 1 kg of charcoal or biochar produced from *Leucaena leucocephala* (Lam.) de Wit costs 20 Baht; if this is applied to activated carbon production, 0.9 kg of product can be obtained. For the modification process, Liu *et al.*^11^ prepared FeCl_3_-modified activated carbon (positive charge/Lewis acid) using an impregnation process. They found that FeCl_3_ could serve as an oxidizing agent to increase the amount of carbonyl groups on the carbon surface, leading to the enhancement of the adsorption ability towards Cr_2_O_7_^2−^ (negative charge/Lewis base) *via* electrostatic forces. Luo *et al.*^13^ reported that even MnO_2_ exhibited good adsorption abilities for heavy metals, but it is still unfavorable for use because of its poor physical–chemical properties. Thus, they developed MnO_2_/Fe_3_O_4_/o-MWCNT as a good candidate adsorbent for the removal of Cr(vi), and the results showed that a maximum Cr(vi) adsorption capacity of 186 mg g^−1^, derived from the Langmuir model, was achieved. Feng *et al.*^14^ tried to activate and modify activated carbon derived from *Astragalus* residue with KOH and KMnO_4_, respectively, for the adsorption of Cd^2+^ from aqueous solution. The maximum Cd^2+^ adsorption capacities of the activated carbon before and after KMnO_4_ modification were 116 and 217 mg g^−1^, respectively. They also reported that KMnO_4_ was indicated to be the best oxidizing agent and expected that it could be applied to the adsorption of other metal ions. However, for the preparation processes above, it needs to be calcined at very high temperatures, which requires a huge amount of energy. Moreover, complex technology is required. Thus, developing an environmentally friendly modification process with the use of simple technology, as well as controlled production costs, is necessary.^15^ Also, the adsorption capacities and uptake abilities should be improved from current values.

Based on the above discussion, the abundant existence of carbonyl groups on the surface of biochar produced from a carbonization process in a confined space needs further modification through the functionalization of carboxylic groups on the surface for the better adsorption of cations. It should be noted that a harsh oxidizing agent is required due to the specific structure of charcoal. Considering economic factors, the objectives of this work are to improve and develop the performance of low-cost biochar for the selective adsorption of multiple metals with high capacities. Here, palm kernel cake (biomass residue derived from the palm oil production process) was applied as a feedstock for biochar-adsorbent production. The surface modification of the as-prepared biochar was investigated using oxidizing agents such as HNO_3_ and KMnO_4_ at different ratios. The adsorption ability of each prepared adsorbent was compared with commercial activated carbon through considering the adsorption capacities for metal cations and iodine number values. The optimum conditions, such as the pH value, ion concentration and adsorbent amount, and the reusability were investigated in detail. The physical and chemical properties of the as-prepared adsorbents, such as BET surface area, functional group composition, acidity/basicity, morphology, thermal decomposition properties and pH_pzc_, were studied. To obtain more details regarding the adsorption behavior, various parameters, such as the surface chemistry, isotherms, kinetic models, and intra-particle diffusion and thermodynamic data, were determined systematically. To the best of our knowledge, there are no reports in the literature on the use of this kind of low-cost adsorbent for the selective removal of Fe^3+^, Fe^2+^, Ca^2+^ and Zn^2+^ along with studies of its respective adsorption behavior. It is expected that the developed low-cost adsorbent could be applied to metal removal from wastewater from industrial processes.

## 2. Experimental

### 2.1. Materials and reagents

Palm kernel cake was obtained from the local palm oil industry in Thailand, and it was carbonized at 350 °C under confined conditions until no smoke appeared during the carbonization process. The carbonized carbon samples obtained from the above-mentioned method are denoted as CP. Here, CP was crushed and sieved through a 400 mesh sieve before any activation or modification processes. Commercial activated carbon (ACC) was purchased from Sigma-Aldrich. All chemical reagents used were of analytical grade and purchased from Merck and Sigma-Aldrich. Stock solutions (1000 mg L^−1^) of Fe^2+^, Fe^3+^, Ca^2+^ and Zn^2+^ were prepared *via* dissolving FeSO_4_·7H_2_O, FeCl_3_·6H_2_O, CaCl_2_ and ZnCl_2_ in deionized water, respectively.

### 2.2. Adsorbent preparation and modification

In brief, the adsorbent was prepared and modified as follows:

(I) Physical activation: CP was heated at 400–600 °C for 1–5 h. Here, it should be mentioned that, for instance, CP activated at 500 °C for 1 h was denoted as CP501.

(II) Chemical activation (HNO_3_): 5 g of CP was mixed with 50 mL of conc. HNO_3_ and refluxed at 100 °C for 4 h. The samples obtained from this process are denoted as CPHNO_3_.

(III) Chemical activation (KMnO_4_): 5 g of CP was mixed with 50 mL of 0.02 mol L^−1^ KMnO_4_ and stirred at ambient temperature for 4 h. The samples obtained from this process are denoted as CPMn.

(IV) Chemical activation (HNO_3_ + KMnO_4_): 5 g of CP was mixed with 50 mL of 0.02 mol L^−1^ KMnO_4_ and stirred at ambient temperature for 4 h. The samples obtained from this process are denoted as CPMix.

Details of the adsorbent characterization methods carried out (*e.g.*, BET, pH_pzc_, FT-IR, Boehm titration, TG-DTG, XRD and SEM-EDS studies) are provided in the ESI.†^16^

### 2.3. Adsorption studies

All the adsorbents prepared using the above-mentioned method were primarily investigated to find the optimum conditions for the adsorption of Fe^2+^, Fe^3+^, Ca^2+^ and Zn^2+^ before further studies of isotherms, kinetic models and thermodynamic adsorption. The metal cation adsorption procedure was as follows: 0.1 g of adsorbent was added to 25 mL of 250 mg L^−1^ Fe^2+^, 250 mg L^−1^ Fe^3+^, 25 mg L^−1^ Ca^2+^ or 100 mg L^−1^ Zn^2+^ and stirred at a speed of 150 rpm at a temperature of 303.15 K for 30 min. Meanwhile, studies of the adsorption of 0.05 mol L^−1^ I_2_ were also carried out under the same conditions as the metal cation adsorption process. After finishing the processes, the adsorbents were separated *via* filtration and the obtained solutions were then analyzed to find the remaining concentrations of Fe^2+^, Fe^3+^, Ca^2+^, Zn^2+^ and I_2_. The concentrations of Fe^2+^ and Fe^3+^ were analyzed using a 1,10-phenanthroline method with a UV-visible spectrophotometer at a wavelength of 510 nm (Genesys 20), while the Zn^2+^ concentration was analyzed using a flame atomic absorption spectrometer (Thermo Scientific iCE 3000). The concentrations of Zn^2+^ and I_2_ were determined *via* titration with EDTA and S_2_O_3_^2−^ solutions, respectively. The amount of adsorption at equilibrium (*q*_e_, mg g^−1^) was calculated according to eqn (1):1
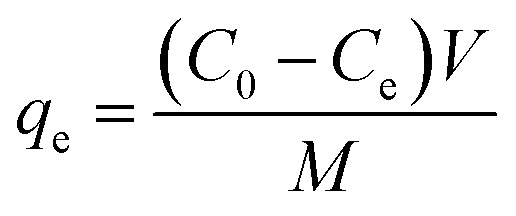
where *C*_e_ is the equilibrium concentration of the adsorbate (mg L^−1^), *C*_0_ is the initial concentration of adsorbate (mg L^−1^), *V* is the volume of solution (L), and *M* is the mass of adsorbent (g).

### 2.4. Investigation of the optimum pH value for Fe^2+^, Fe^3+^, Ca^2+^ and Zn^2+^ adsorption

0.1 g of adsorbent (CPMn) was added to 25 mL of 280 mg L^−1^ Fe^2+^, 210 mg L^−1^ Fe^3+^, 30 mg L^−1^ Ca^2+^ or 75 mg L^−1^ Zn^2+^ and then the pH value was adjusted from 1 to 8 through adding 0.1 mol L^−1^ HCl and 0.1 mol L^−1^ NaOH as required. Thereafter, each mixed solution was stirred at a speed of 150 rpm at a temperature of 303.15 K for 30 min.

### 2.5. Investigation of Fe^2+^, Fe^3+^, Ca^2+^ and Zn^2+^ desorption

0.1 g of adsorbent (CPMn) was added to 5–25 mL of H_2_O, 1.0 mol L^−1^ HNO_3_ or 1.0 mol L^−1^ NaCl and stirred at a temperature of 303.15 K for 30 min at a speed of 150 rpm. Then, the obtained solution was filtrated and analyzed in the presence of desorbed Fe^2+^, Fe^3+^, Ca^2+^ and Zn^2+^.

### 2.6. Investigation of adsorbent reusability

Before reusability testing, used adsorbent (CPMn–Fe^2+^, Fe^3+^, Ca^2+^ or Zn^2+^) was obtained based on the optimum desorption process mentioned above. In short, 0.1 g of regenerated adsorbent (CPMn) was added to 25 mL of 280 mg L^−1^ Fe^2+^, 210 mg L^−1^ Fe^3+^, 30 mg L^−1^ Ca^2+^ or 75 mg L^−1^ Zn^2+^ and stirred at a speed of 150 rpm at a temperature of 303.15 K for 30 min.

### 2.7. Investigation of adsorption isotherms, kinetics and thermodynamics

The details relating to adsorption isotherms, kinetics and thermodynamics are provided in the ESI,†Fig. 7–10 and Tables 2–4.^20–25^

## 3. Results and discussion

### 3.1. Adsorption ability and behavior

**Fig. 1:**
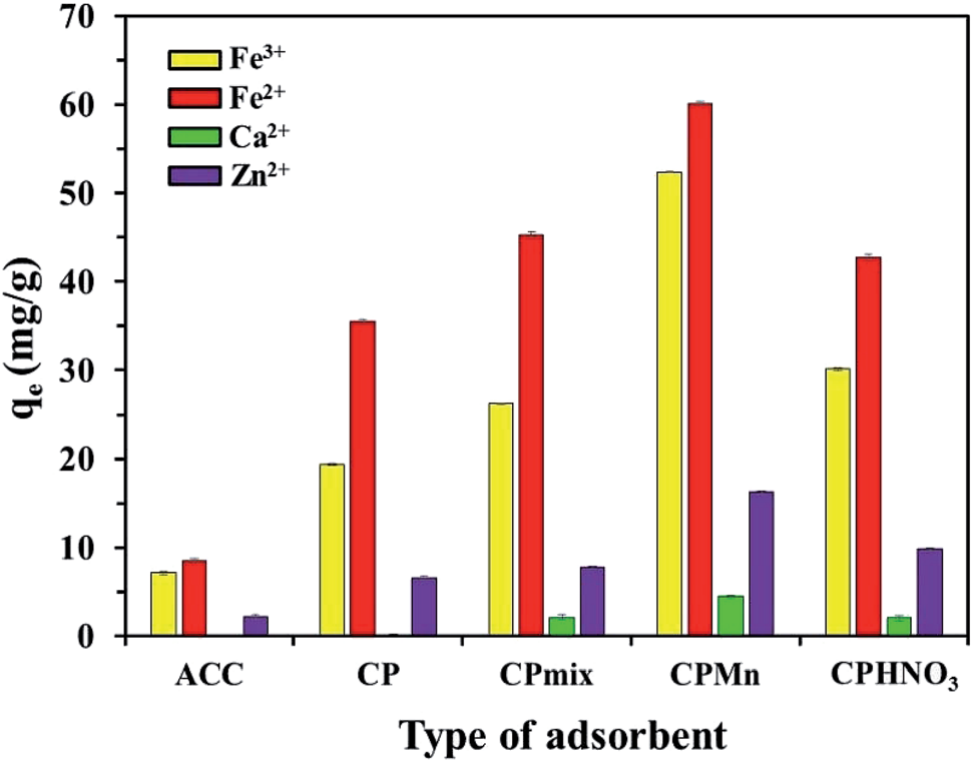
A comparison of the Fe^3+^, Fe^2+^, Ca^2+^ and Zn^2+^ adsorption efficiencies using various adsorbents before and after modification with KMnO_4_ and HNO_3_.

Fig. S1† shows the burn loss percentage results of biochar at different temperatures. As expected, increasing the calcination temperature or time obviously promoted the burn loss of the biochar. The results of I_2_ and Fe^2+^ adsorption using the various adsorbents prepared at different temperatures are shown in Fig. S2.† It is found that ACC provided maximum I_2_ adsorption of 1435 mg g^−1^, suggesting that the surface area, porosity and non-polarity of the surface of ACC was much higher when compared with CP. In contrast, the lowest Fe^2+^ adsorption of 8.56 mg g^−1^ was also found for ACC, while all types of CP exhibited better Fe^2+^ adsorption capacities. This phenomenon could be attributed to the existence of high levels of carbon with low oxygen amounts on the ACC surface, resulting in the excellent adsorption of non-polar I_2_ molecules.^6^ In the case of the adsorption behavior of Fe^2+^, CP had high levels of oxygen-containing functional groups, such as carbonyl groups with lone electron pairs (Lewis base), leading to the good adsorption of Fe^2+^ cations (Lewis acid) *via* attraction through electrostatic forces with the generation of co-ordinate covalent bonds. It should be noted that increasing the calcination temperature and time resulted in a slight reduction in Fe^2+^ adsorption capacity, while the I_2_ adsorption capacity was increased to some extent; this was probably due to the decomposition of carbonyl groups on the adsorbent surface into CO_2_, leading to a decrease in the polarity of the adsorbent structure. Thus, CP without physical activation was selected for further study. Fig. 1 shows the results of Fe^3+^, Fe^2+^, Ca^2+^ and Zn^2+^ adsorption using various adsorbents, including those modified with KMnO_4_ and HNO_3_. Here, CP could be directly modified with KMnO_4_ and Fe^3+^ without pyrolysis activation, which could greatly reduce the production costs of adsorbents. Interestingly, one can clearly see that CPMn exhibited the highest adsorption performance towards Fe^3+^, Fe^2+^, Ca^2+^ and Zn^2+^, followed by CPHNO_3_. This should result from the effects of modification with KMnO_4_ and HNO_3_ through which the functional groups on the CP structure were changed into carbonyl/carboxylic groups as per the following equations:

2 –C–C–C–OH + KMnO_4_ → –C–C

<svg xmlns="http://www.w3.org/2000/svg" version="1.0" width="13.200000pt" height="16.000000pt" viewBox="0 0 13.200000 16.000000" preserveAspectRatio="xMidYMid meet"><metadata>
Created by potrace 1.16, written by Peter Selinger 2001-2019
</metadata><g transform="translate(1.000000,15.000000) scale(0.017500,-0.017500)" fill="currentColor" stroke="none"><path d="M0 440 l0 -40 320 0 320 0 0 40 0 40 -320 0 -320 0 0 -40z M0 280 l0 -40 320 0 320 0 0 40 0 40 -320 0 -320 0 0 -40z"/></g></svg>

O + –C–COOH + MnO_2_

3 –C–C–C–OH + –C–C–C– + HNO_3_ → –C–CO + –C–COOH

Considering the cation sizes (Ca^2+^ (231 pm) > Fe^2+^ (76 pm) > Zn^2+^ (74 pm) > Fe^3+^ (64 pm)) and *E*^0^ values (Ca^2+^ (−2.869 V) < Zn^2+^ (−0.76 V) < Fe^2+^ (−0.44 V) < Fe^3+^ (−0.04 V)), the Ca^2+^ cation was found to be most difficult to adsorb when compared with the other cations. Also, CP and ACC without any modification could not adsorb Ca^2+^ from aqueous solution. This suggests that the carboxylic groups on CP modified with KMnO_4_ and HNO_3_ and MnO_2_ formation on the surface of CP occurring from the reduction of KMnO_4_ were required for the Ca^2+^ adsorption mechanism, as well as for the adsorption of other cations. Here, comparing the highest adsorption capacities of all types of studied cations, Fe^2+^ was found to be much more easily adsorbed with CPMn (60.18 mg g^−1^). It should be mentioned here that even though the modification of CP with HNO_3_ was successfully achieved, lower adsorption performance towards various metal cations was still found compared with CP modified with KMnO_4_. Moreover, it requires a large amount of water for the washing procedure and pH adjustment. Therefore, we proposed to use KMnO_4_ for adsorbent modification, which presented low-production costs and a non-complicated process. In order to get more detail, the effects of various ratios of CP to KMnO_4_ or HNO_3_ on I_2_ and Fe^2+^ adsorption are shown in Fig. S3.† It is found that the I_2_ adsorption capacity was increased to some extent upon only increasing the ratio of CP to HNO_3_. Highest I_2_ adsorption capacities of 866.62 mg g^−1^ and 689.71 mg g^−1^ were found for CPHNO_3_ 1 : 50 and CPMn 1 : 10, respectively. Also, CPMn 1 : 10 provided a maximum Fe^2+^ adsorption capacity. Increasing the KMnO_4_ or HNO_3_ amount had no significant effect on improving the Fe^2+^ adsorption efficiency. Here, KMnO_4_ was presented to be a better oxidizing agent than HNO_3_, probably due to the formation of MnO_2_ crystals, which promoted the adsorption capabilities for metal cations. Based on the above results, since CPMn had better performance for Fe^3+^, Fe^2+^, Ca^2+^ and Zn^2+^ adsorption, CPMn with an optimum ratio of CP to KMnO_4_ (1 : 10) was selected for further studies, such as investigating the effects of pH, desorption and reusability, as well as equilibrium adsorption.

### 3.2. Characterization of the as-prepared adsorbent

**Fig. 2:**
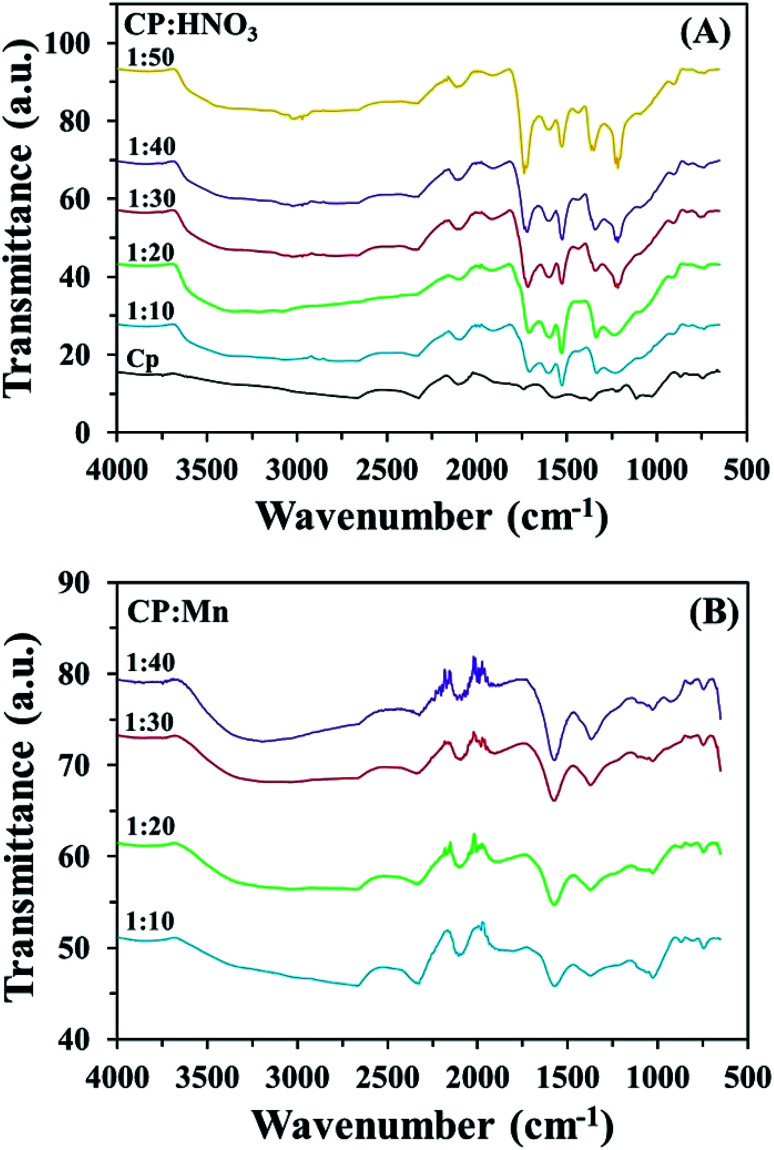
FT-IR spectra of CP before and after modification with (A) HNO_3_ and (B) KMnO_4_ at different ratios of CP to HNO_3_ or KMnO_4_.

Table 1 shows the surface chemical properties of CP, CPHNO_3_ and CPMn. As obtained, the pH_pzc_ values of all adsorbents had higher values than the pH of the mixed solution (adsorbent + each cation: Fe^3+^, Fe^2+^, Ca^2+^ and Zn^2+^), indicating that the surfaces of all adsorbents were occupied by negative charge (pH < pH_pzc_), resulting in the high Fe^3+^, Fe^2+^, Ca^2+^ and Zn^2+^ adsorption abilities. The acidity and basicity levels of CP, CPHNO_3_ and CPMn are shown in Table 1. It is found that CP modified with HNO_3_ (CPHNO_3_) and KMnO_4_ (CPMn) had more carboxylic groups than CP without modification. It should be noted that the basicity of the adsorbent was obviously increased in the case of CPMn. This phenomenon could be generally attributed to the formation of a brown precipitate of MnO_2_, which could react with HCl leading to an increase in the adsorbent basicity. Moreover, from checking the results of the physical properties of each adsorbent, CPHNO_3_ and CPMn had higher surface areas than CP (Table 1). This might be one reason for the improved adsorption capacities for metal cations. A highest surface area of 56 m^2^ g^−1^ was found for CPMn, which had a surface area more than nine times that of CP. This should result from the MnO_2_ contribution, which was obtained from the modification of CP by KMnO_4_*via* an *in situ* reduction process. FT-IR spectra of CP calcined at different temperatures and times are shown in Fig. S4.† As observed, CP calcined at 400–600 °C for 1–4 h presented peaks at wavenumbers of 3500 cm^−1^ (–OH and –NH), 2900 cm^−1^ (C–H), 1735 cm^−1^, 1600 cm^−1^ (C=O and C=C), 1217 cm^−1^, 1114 cm^−1^ and 1030 cm^−1^ (C–O), which should be attributed to the vibrations of various acid–base functional groups, such as carboxylic, lactone, phenolic and amino groups.^17^ Here, the calcination temperature and time had significant effects on the enhancement of carbonyl group levels. However, the decomposition of these functional groups on activated CP may occurred with conversion into CO_2_ in the case of excessively high calcination temperatures and long calcination times being applied, leading to deactivation and the low performance of the adsorbent for metal cation removal. In addition, C–O vibrations at a wavenumber of 1030 cm^−1^ were found for CP calcined at 600 °C for 4 h (CP604). This could be explained as resulting from the decomposition of CP into ash upon exceeding the temperature for the combustion process. Interestingly, as shown in Fig. S5,† the wavenumbers of the oxygen functional group peaks from the CPHNO_3_ structure at 1735 cm^−1^ (C=O), 1550 cm^−1^ (–COO–), 1250 cm^−1^ (–C–O) and 3450 cm^−1^ (–OH) strongly increased and presented higher vibrational intensities than CP, CPMn and CPMix, which could be assigned to the high amount of carboxylic groups on the surface (Rios *et al.*, 2013).^18^ Moreover, spectra from CP modified with HNO_3_ and KMnO_4_ at different ratios of CP to HNO_3_ or KMnO_4_ are also shown in Fig. 2. An increase in the ratio of CP to HNO_3_ from 1 : 10 to 1 : 50 and ratio of CP to KMnO_4_ from 1 : 10 to 1 : 40 resulted in obvious increases in the carbonyl group peaks at wavenumbers of 1730 and 1600 cm^−1^, respectively, on the surface of modified CP. Here, the optimum ratios of CP to HNO_3_ and KMnO_4_ were 1 : 50 and 1 : 40, respectively.

**Table:** Physicochemical properties of the as-prepared adsorbents

Adsorbent	BET surface area (m^2^ g^−1^)	Amount of functional groups (*m*_eq_/g)						pH_pzc_	pH in Fe^3+^	pH in Fe^2+^	pH in Ca^2+^	pH in Zn^2+^
		Carboxylic	Lactonic	Phenolic	Total acidic	Total basic	Total acidic and basic					
CP	6.4	0.0739	0.0362	4.0273	4.1374	2.2587	6.3961	9.14	4.63	2.68	6.60	7.95
CPHNO_3_	14.7	5.5360	0.0347	0.4250	5.9957	0.2751	6.2708	3.45	3.22	2.93	2.98	3.29
CPMn	56.2	0.1658	0.0398	0.4436	0.6492	7.1817	7.8309	9.23	5.39	3.25	7.54	3.89

**Fig. 3:**
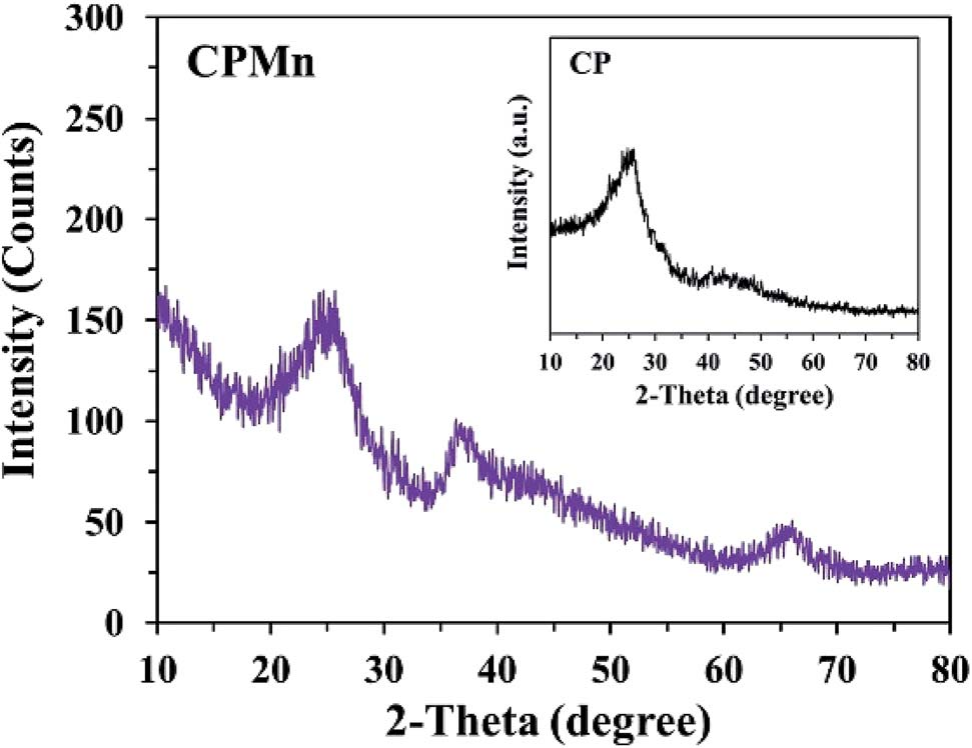
The XRD pattern of CP modified with KMnO_4_. The XRD pattern of CP without modification is shown in the inset.

**Fig. 4:**
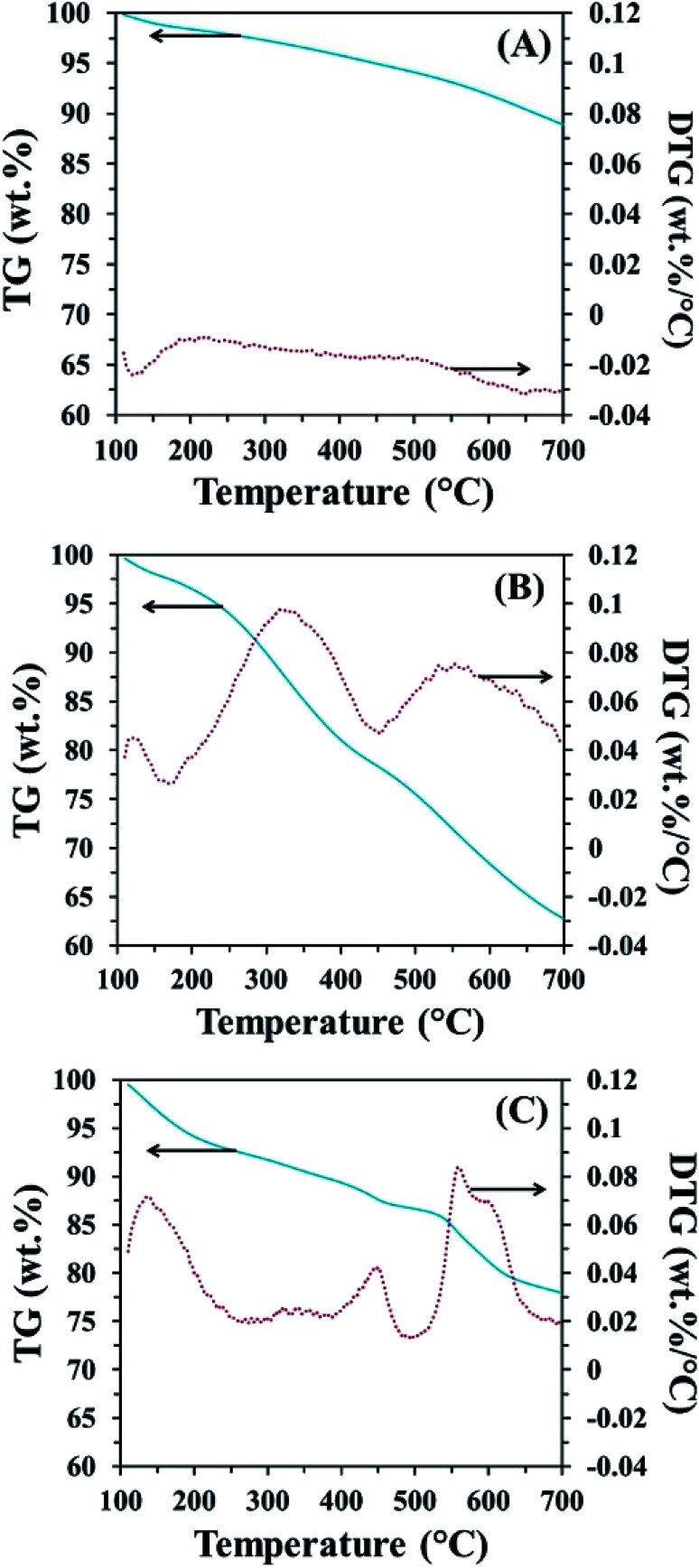
TG-DTG profiles of (A) CP, (B) CPHNO_3_ and (C) CPMn.

**Fig. 5:**
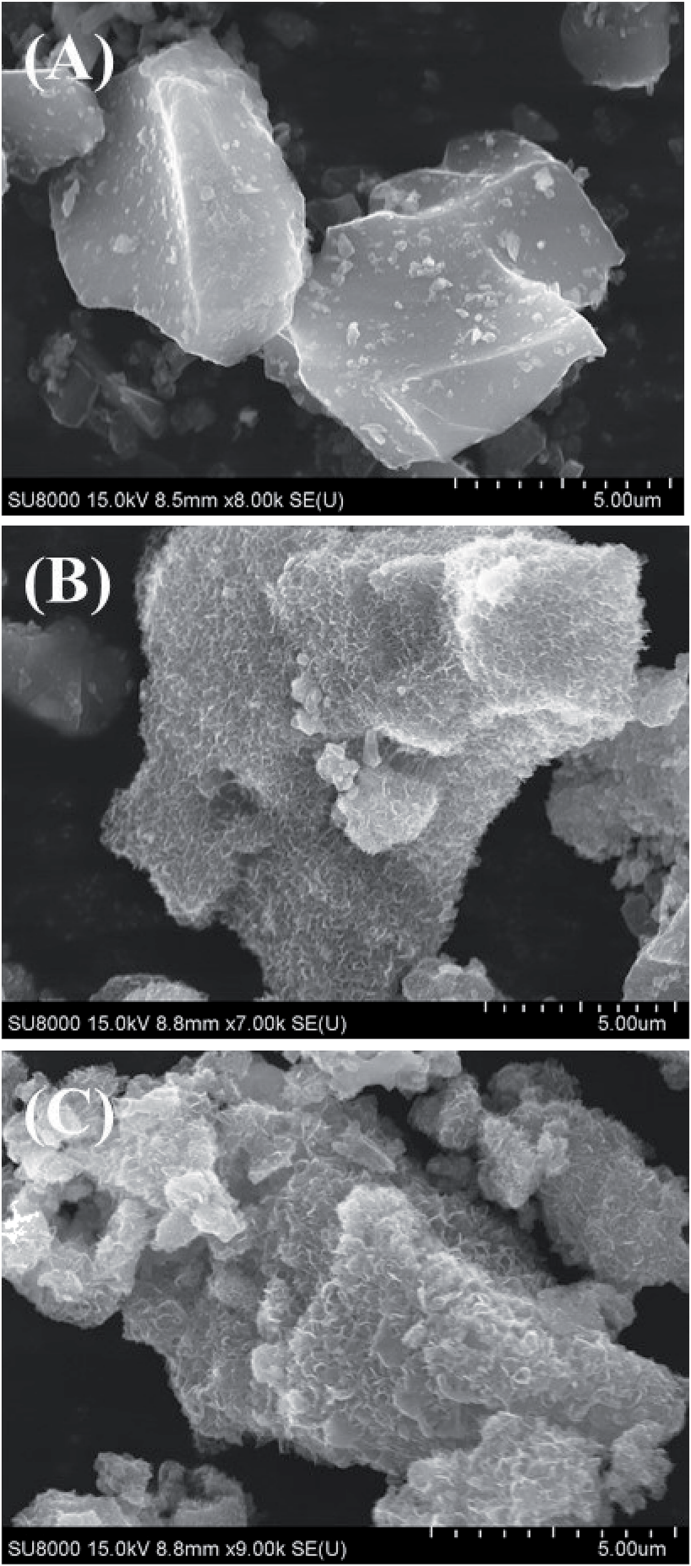
SEM images of (A) CP, (B) CPMn and (C) CPMn after the Fe^3+^ adsorption process.

In order to confirm the existence of MnO_2_ on the CP surface after modification with KMnO_4_ together with an *in situ* reduction process, the XRD pattern of CPMn, as shown in Fig. 3, clearly exhibits diffraction peaks from MnO_2_ at 2-theta values of 29° (310), 38° (211) and 40° (301), indicating that MnO_2_ was obviously formed *via* KMnO_4_ reduction.^14^Fig. 4 shows TG-DTG profiles with the thermal decomposition data from various adsorbents. Moisture/water evaporation from the structures was found for all adsorbents at a thermal decomposition temperature of about 100 °C, while the carboxylic groups decomposed at 200–650 °C.^18^ In the case of CPHNO_3_, the thermal decomposition peaks of the carboxylic groups clearly appeared at 200–400 °C and 400–650 °C. It should be mentioned here that a higher decomposition temperature of 450–650 °C was found for CPMn, which might be attributed to the decomposition of carbonyl groups occurring from oxidation by KMnO_4_. The morphologies of the adsorbents CP, CPMn and CPMn after the adsorption of metal cations, such as Fe^3+^, were observed *via* SEM (Fig. 5). As seen, the surface morphology of CP was smooth, whereas that of CPMn was rough and uneven (curved petal-like walls). After the cation metal adsorption process, no significant change in the surface morphology of CPMn was found in this study. Also, the presence of metal species was also observed *via* SEM-EDS (Fig. S6†). No bulk MnO_2_ particles were found on the modified CP, suggesting that the MnO_2_ particles were of very small size and were well dispersed on the adsorbent surface. Moreover, the existence of metal species such as Mn, Fe, Ca and Zn was also confirmed after the adsorption process was carried out.

### 3.3. Effects of pH on Fe^3+^, Fe^2+^, Ca^2+^ and Zn^2+^ adsorption

**Fig. 6:**
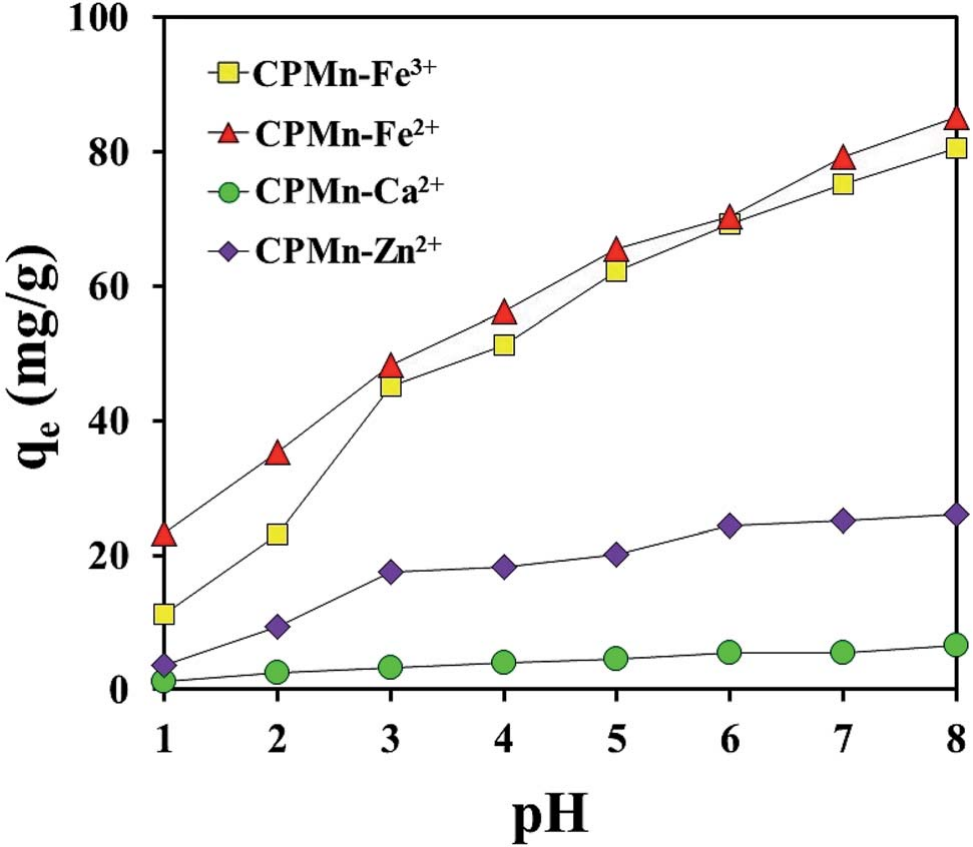
The effects of pH on the adsorption of Fe^3+^, Fe^2+^, Ca^2+^ and Zn^2+^ by CP modified with KMnO_4_.

Fig. 6 shows the effects of pH on Fe^3+^, Fe^2+^, Ca^2+^ and Zn^2+^ adsorption using CPMn. The ability of CPMn to adsorb metal cations increased with an increase in solution pH. In the case of solutions with a low pH value, a large amount of H_3_O^+^ should exist, which could possibly result in interference in the system, leading to the low adsorption efficiency of CPMn. It should be mentioned here that at a low pH value, H_3_O^+^ might react with MnO_2_ (basic) existing on CP, resulting in a reduction of the adsorption capacity towards metal cations. In other words, as the surface negative charge density of CPMn reduced with a decrease in the pH value, electrostatic repulsion between the positively charged metal cations and the surface of the adsorbents was also inflated, which may result in a decrease in the adsorption capacity. Moreover, it is also found that when the pH value in solution was >7, the adsorption capacities for Fe^3+^, Fe^2+^, Ca^2+^ and Zn^2+^ continuously increased. This suggests that these ions possibly could react with OH^−^ to form Fe(OH)_3_, Fe(OH)_2_, Ca(OH)_2_ and Zn(OH)_2_ precipitates at pH > 7. Thus, the high Fe^3+^, Fe^2+^, Ca^2+^ and Zn^2+^ adsorption abilities at pH > 7 did not result from using CPMn. Here, the adsorption capacities for each metal cation in this study were quite different, probably due to the different effects of ion size, stability and other factors. Based on these results, it is not necessary to adjust the pH value of ion solutions when using CPMn as an adsorbent for further studies.

### 3.4. Effects of Fe^3+^, Fe^2+^, Ca^2+^ and Zn^2+^ desorption

**Fig. 7:**
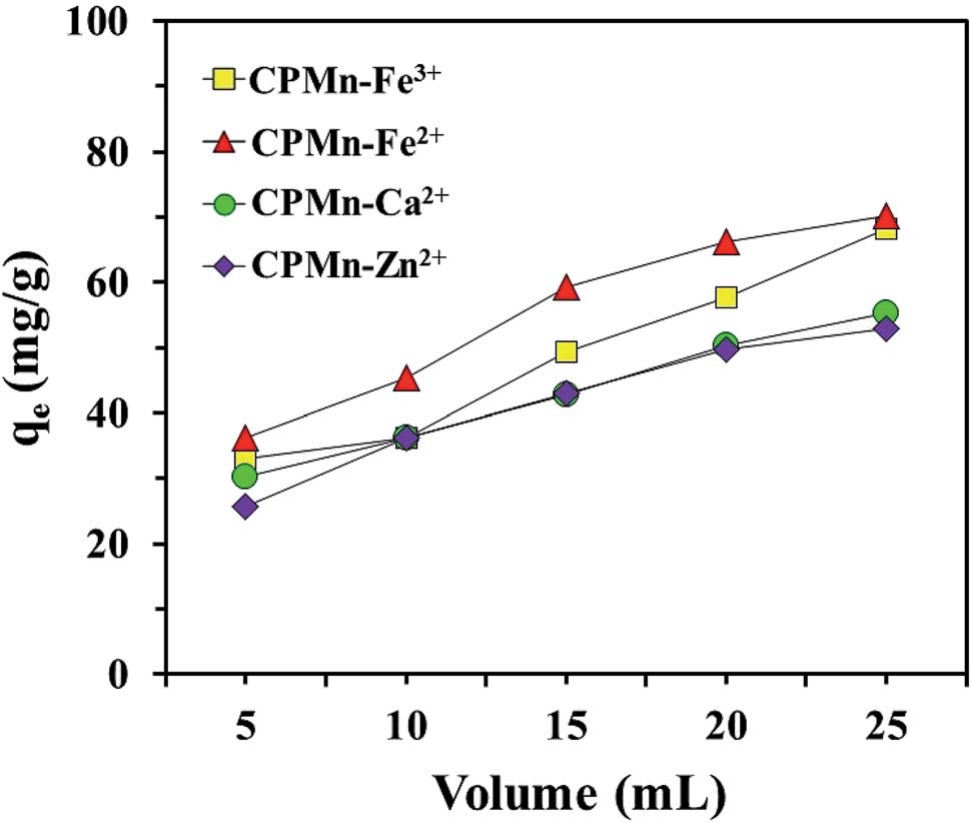
The desorption of Fe^3+^, Fe^2+^, Ca^2+^ and Zn^2+^ from CP modified with KMnO_4_ using different volumes of 1.0 mol L^−1^ HNO_3_.

Fig. 7 shows the desorption efficiencies of Fe^3+^, Fe^2+^, Ca^2+^ and Zn^2+^ using CPMn. Increases in the desorption percentages of Fe^3+^, Fe^2+^, Ca^2+^ and Zn^2+^ were graphically observed when the volume of 1.0 mol L^−1^ HNO_3_ was increased, suggesting that the presence of HNO_3_ in high amounts could demolish the force of attraction between functional groups with lone pairs of electrons (Lewis base) and the four metal cations (Lewis acids). This indicates that spent adsorbent could be easily regenerated upon washing with HNO_3_ solution. However, it is also possible that the simultaneous desorption of metal cations and anchored MnO_2_ from the adsorbent surface occurs in the case of using high concentrations of HNO_3_. In the case of pure water and 1.0 mol L^−1^ NaCl, the results are not shown here since no desorption of Fe^3+^, Fe^2+^, Ca^2+^ and Zn^2+^ from CPMn was significantly detected, even though the amounts of pure water and 1.0 mol L^−1^ NaCl were adjusted. This indicates that the attraction forces between the metal cations and CPMn were quite strong. Therefore, HNO_3_ was chosen as a suitable chemical for application to Fe^3+^, Fe^2+^, Ca^2+^ and Zn^2+^ desorption.

### 3.5. Reusability of the adsorbents

**Fig. 8:**
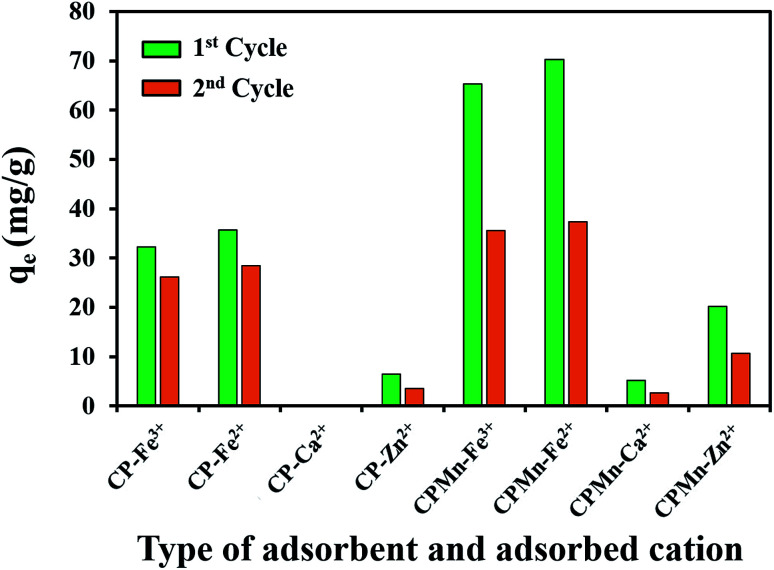
Reusability of CP modified with KMnO_4_ for the adsorption of Fe^3+^, Fe^2+^, Ca^2+^ and Zn^2+^.

In this study, spent CPMn was regenerated by using 1.0 mol L^−1^ HNO_3_ for Fe^3+^, Fe^2+^, Ca^2+^ and Zn^2+^ desorption, and it was reused again under the same conditions (Fig. 8). It is found that after regenerated CPMn was applied to Fe^3+^, Fe^2+^, Ca^2+^ and Zn^2+^ adsorption in a second cycle, the adsorption capacity for each metal cation decreased by about 45% when compared with the fresh sample. Also, this can be compared with regenerated CP without KMnO_4_ modification, where only an 18% reduction was found during reusability testing. This phenomenon could be explained based on the reaction of HNO_3_ with MnO_2_ on CP during the regeneration process. From these results, it can be seen that CPMn is not suitable for reuse. Moreover, during the general regeneration of spent adsorbent, strong acid and a lot of water is required for the washing procedure, leading to an increase in the production costs of the adsorbent. However, considering the production costs of biochar from palm kernel cake or other agricultural waste biomass, the normal prices of biochar/carbonization (0.6 USD per kg), grinding (0.6 USD per kg) and KMnO_4_ chemical activation (4.0 USD) and the overall cost (6.3 USD per kg) were found to be much cheaper than that of commercial activated carbon (127 USD per kg). Meanwhile, CPMn also exhibited better performance than commercial activated carbon for metal cation adsorption. Thus, it is not necessary to regenerate/reuse it *via* washing with acid solution.

### 3.6. Adsorption equilibrium

**Fig. 9:**
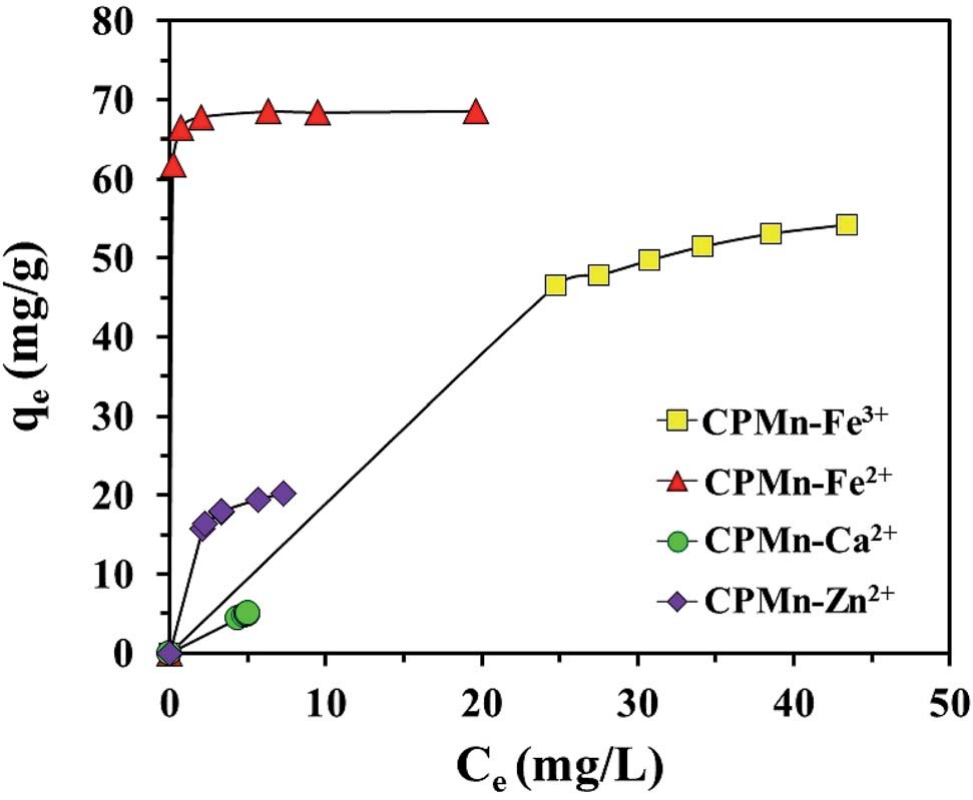
The equilibrium adsorption of Fe^3+^, Fe^2+^, Ca^2+^ and Zn^2+^ by CP modified with KMnO_4_. The relative combined expanded uncertainty tSE is <±0.03 mg g^−1^ (0.95 level of confidence). The initial concentration of adsorbate (*C*_0_): Fe^3+^ = 210–260 mg L^−1^, Fe^2+^ = 200–250 mg L^−1^, Ca^2+^ = 20–30 mg L^−1^, Zn^2+^ = 65–90 mg L^−1^; 25 mL; adsorbent amount = 0.10 g; time (*t*) = 60 min; temperature (*T*) = 303.15 K; pressure (*P*) = 101 kPa.

In this study, the adsorption behavior was studied; six mathematical isotherm models, the Langmuir, Freundlich, Temkin, Dubinin–Radushkevich, Redlich–Peterson and Toth models, were applied based on varying concentrations of adsorbate ion solution, as shown in Fig. 9. For non-linear methods, a trial/error process, which was reasonable to apply *via* computer operations, was applied to assign the isotherm, kinetic, intra-particle diffusion and thermodynamic parameters to maximize the correlation coefficients obtained from experimental results. In this study, *R*^2^ was used to test the best-fitting isotherm using the equation expressed in eqn (4):^19^4
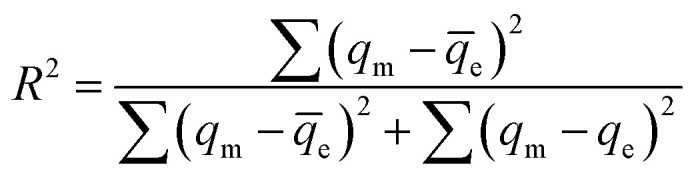
where *q*_m_ (mg g^−1^) is the equilibrium capacity obtained from the isotherm model that was calculated using the Excel solver, *q*_e_ (mg g^−1^) is the equilibrium capacity obtained from experimental data, and *q̄*_e_ (mg g^−1^) is the average of *q*_e_.

The details of each isotherm model and the applied equations are also provided in the ESI.† As shown in Fig. 9, metal adsorption capacities using CPMn were in the order: Fe^2+^ > Fe^3+^ > Zn^2+^ > Ca^2+^. Table 2 shows the equilibrium isotherms calculated *via* non-linear methods for Fe^3+^, Fe^2+^, Ca^2+^ and Zn^2+^ adsorption using CPMn. Considering the *R*^2^ values, the adsorption results for all metal cations using CPMn were well fitted using the Langmuir isotherm model (*R*^2^ > 0.99), and the fits were also found to be greater than those obtained using the Freundlich adsorption isotherm, indicating monolayer adsorption of Fe^3+^, Fe^2+^, Ca^2+^ and Zn^2+^ (Lewis acid) on the CPMn surface *via* chemical attraction through electrostatic force with the generation of co-ordinate covalent bonds. The *q*_max_ values of Fe^2+^, Fe^3+^, Zn^2+^ and Ca^2+^ adsorption using CPMn were 70.67, 68.60, 22.38, and 5.06 mg g^−1^, respectively, which also exhibited the order: Fe^2+^ > Fe^3+^ > Zn^2+^ > Ca^2+^. For the Temkin isotherm model, one can see that only the *R*^2^ values for Fe^3+^ and Zn^2+^ adsorption were close to 1. It should be mentioned here that Zn^2+^ was the most strongly anchored on the surface of CPMn, as verified by it having the highest *A* value (182.83 L mol^−1^) calculated from the Temkin model. The Dubinin–Radushkevich parameters could be considered as useful since suitable *R*^2^ values (>0.9) were found for all metal cations. Here, the *E* values for the adsorption of the metal cations Fe^3+^, Zn^3+^ and Ca^2+^ were lower than 8 kJ mol^−1^, corresponding to a physisorption process.^24^ In the case of Fe^2+^, the *E* value was in the range of 8–16 kJ mol^−1^, which could be attributed to chemisorption behavior between Fe^2+^ and the CPMn surface. Meanwhile, the *q*_s_ value calculated from the Dubinin–Radushkevich isotherm was close to the *q*_max_ value calculated from the Langmuir isotherm, indicating the accuracy of the applied model. Also, the highest value of *q*_s_ using CPMn was found for Fe^2+^ adsorption. This result is in good agreement with those mentioned above. For the Redlich–Peterson and Toth models, they were applied to support the assumptions of the Langmuir isotherm model through considering the *g* constant. As expected, the *g* constant, Th and *R*^2^ values of these models were close to 1, confirming a monolayer adsorption process, especially for CPMn–Fe^2+^, CPMn–Ca^2+^ and CPMn–Zn^2+^. In addition, it is clearly found that a maximum *q*_e_^∞^ value was obtained for Fe^3+^ adsorption, indicating the potential shown by CPMn for metal cation removal in this study.

Langmuir, Freundlich, Temkin and Dubinin–Radushkevich isotherm models and their parameters with correlation coefficients obtained using non-linear methods. The initial concentration of adsorbate (*C*_0_): Fe^3+^ = 210–260 mg L^−1^, Fe^2+^ = 200–250 mg L^−1^, Ca^2+^ = 20–30 mg L^−1^, Zn^2+^ = 65–90 mg L^−1^; 25 mL; adsorbent amount = 0.10 g; time (*t*) = 60 min; temperature (*T*) = 303.15 K; pressure (*P*) = 101 kPa. All parameters were calculated *via* nonlinear regression in the Excel program

**Table:** 

Adsorbent	Ion type	Langmuir parameters			Freundlich parameters		
		*q*_max_ (mg g^−1^)	*K* (L mg^−1^)	*R* ^2^	1/*n*	*K*_F_	*R* ^2^
CPMn	Fe^3+^	70.67	0.08	0.9956	0.28	18.86	0.9897
	Fe^2+^	68.60	46.32	0.9989	0.02	65.64	0.7758
	Ca^2+^	5.06	13.51	0.9964	0.04	4.66	0.8460
	Zn^2+^	22.38	2.31	0.9942	0.16	16.11	0.9789

**Table:** 

Adsorbent	Ion	Temkin parameters			Dubinin–Radushkevich parameters			
		*b*	*A* (L mol^−1^)	*R* ^2^	*q*_s_ (mg g^−1^)	*B* (mol^2^ kJ^−1^)	*E* (kJ mol^−1^)	*R* ^2^
CPMn	Fe^3+^	173.25	1.04	0.9929	58.52	2.48 × 10^−5^	0.20	0.9885
	Fe^2+^	1861.38	2.57 × 10^21^	0.7883	68.42	5.86 × 10^−9^	13.07	0.9761
	Ca^2+^	11 794.43	4.18 × 10^9^	0.8599	5.01	1.85 × 10^−8^	7.35	0.9925
	Zn^2+^	807.54	182.83	0.9890	20.62	9.93 × 10^−8^	3.17	0.9517

**Table:** 

Adsorbent	Ion	Redlich–Peterson parameters				Toth parameters			
		*A*	*B*	*g*	*R* ^2^	*q*_e_^∞^	*K*_Th_	Th	*R* ^2^
CPMn	Fe^3+^	1113.70	58.80	0.72	0.9897	77.38	6.096	0.784	0.9950
	Fe^2+^	3266.69	47.69	1.00	0.9992	68.67	0.022	0.939	0.9994
	Ca^2+^	59.91	11.68	1.01	0.9991	5.03	0.076	1.195	0.9992
	Zn^2+^	71.38	3.53	0.95	0.9984	24.45	0.340	0.668	0.9987

### 3.7. Adsorption kinetics and intra-particle diffusion

**Fig. 10:**
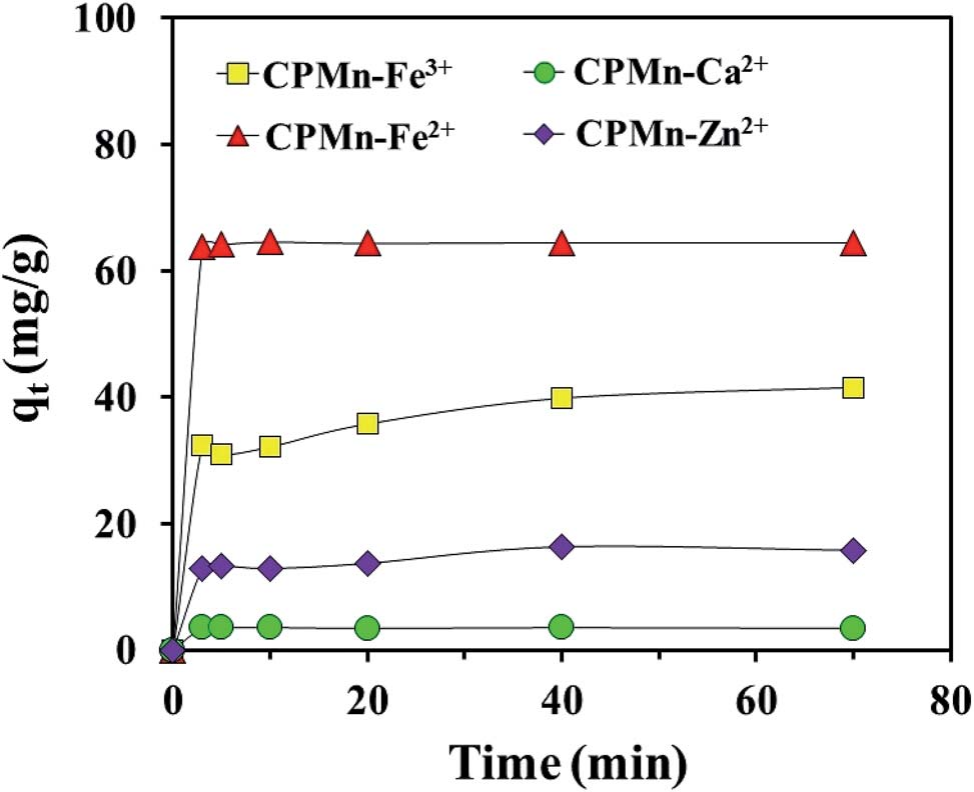
Effect of contact time on the adsorption of Fe^3+^, Fe^2+^, Ca^2+^ and Zn^2+^ by CP modified with KMnO_4_. The relative combined expanded uncertainty tSE is <±0.02 mg g^−1^ (0.95 level of confidence). The initial concentration of adsorbate (*C*_0_): Fe^3+^ = 210 mg L^−1^, Fe^2+^ = 280 mg L^−1^, Ca^2+^ = 20 mg L^−1^, Zn^2+^ = 75 mg L^−1^; 25 mL; adsorbent amount = 0.10 g; time (*t*) = 3–70 min; temperature (*T*) = 303.15 K; pressure (*P*) = 101 kPa.

**Fig. 11:**
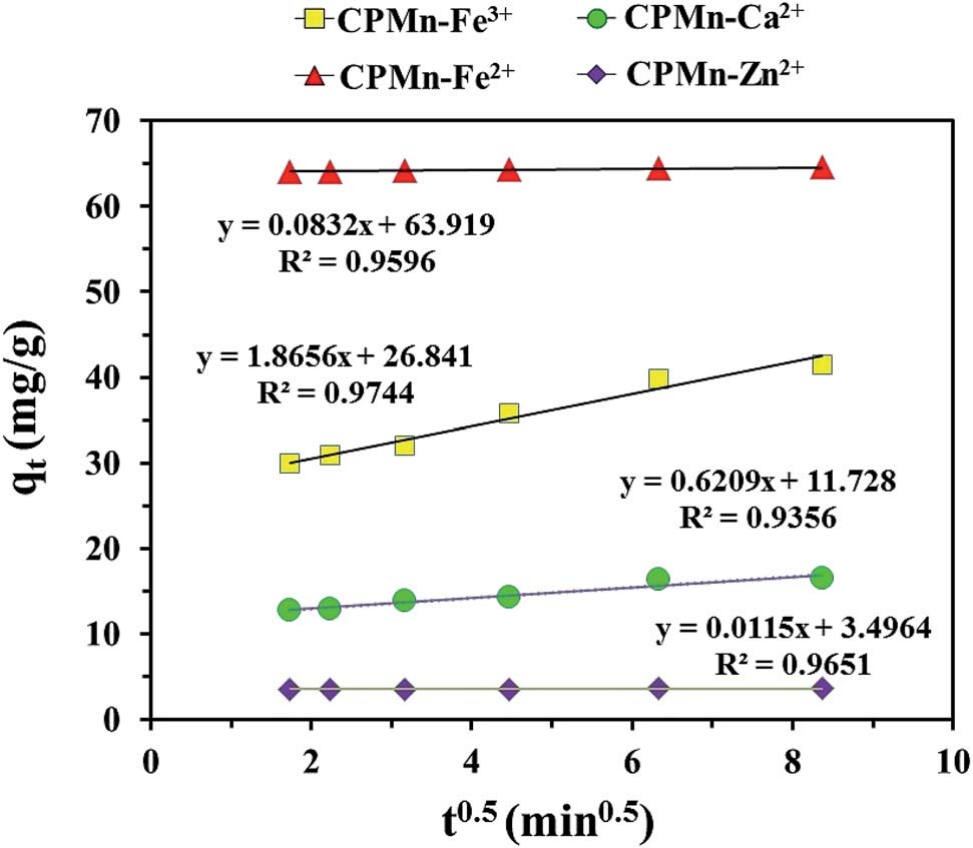
Intra-particle diffusion plots for Fe^3+^, Fe^2+^, Ca^2+^ and Zn^2+^ adsorption onto CPMn. The relative combined expanded uncertainty tSE is <±0.02 mg g^−1^ (0.95 level of confidence).

Fig. 10 show the adsorption capacities of Fe^3+^, Fe^2+^, Ca^2+^ and Zn^2+^ after different contact times using CPMn. Here, rapid initial adsorption was shown after a contact time of 3 min, with the same trend for all adsorbed cations; then adsorption gradually increased and became close to equilibrium within 40 min. This phenomenon might be explained based on the existence of many adsorption sites with adequate vacant sites present during the inception phase. In addition, the absorption site saturation point of CPMn for each metal cation was easily reached as the time extended. To obtain more details regarding the adsorption behavior, as shown in Table 3, the Fe^3+^, Fe^2+^, Ca^2+^ and Zn^2+^ adsorption mechanisms on CPMn were studied and found to be controlled by pseudo second-order kinetics, based on the greater *R*^2^ coefficient (*R*^2^ > 0.99) compared to pseudo first-order kinetics, indicating a very fast adsorption process. Meanwhile, the calculated *q*_e_ values were also very close to the *q*_e_ values derived from the experimental results, suggesting that this model has high accuracy. In addition, the experimental observations were further investigated *via* intra-particle diffusion using the Weber–Morris model, as given by eqn (5),^26^ and the results are shown in Fig. 11.

5 *q*_*t*_ = *k*_p_*t*^0.5^

where *k*_p_ (mg g^−1^ min^0.5^), the intraparticle diffusion rate constant, is obtained from the slope of the straight-line plot of *q*_*t*_*versus t*^0.5^.

**Table:** Pseudo-first-order and pseudo-second-order kinetic model parameters. The initial concentration of adsorbate (*C*_0_): Fe^3+^ = 210 mg L^−1^, Fe^2+^ = 280 mg L^−1^, Ca^2+^ = 20 mg L^−1^, Zn^2+^ = 75 mg L^−1^; 25 mL; adsorbent amount = 0.10 g; time (*t*) = 3–70 min; temperature (*T*) = 303.15 K; pressure (*P*) = 101 kPa.^a^ All parameters were calculated *via* nonlinear regression in the Excel program

Adsorbent	Ion type	*q*_e exp_ (mg g^−1^)	Pseudo-first-order			Pseudo-second-order		
			*q*_e cal_	*k*_1_	*R* ^2^	*q*_e cal_	*k*_2_	*R* ^2^
CPMn	Fe^2+^	64.50	0.14	0.005	0.0042	64.44	0.438	1.0000
	Fe^3+^	43.45	28.18	0.046	0.8905	43.55	0.008	0.9987
	Ca^2+^	3.55	0.02	−0.023	0.3070	3.44	0.550	0.9991
	Zn^2+^	16.40	3.33	0.043	0.3859	16.29	0.190	0.9971

a *q*_e_ is the amount of adsorption at equilibrium; the relative combined expanded uncertainty tSE is <±0.03 mg g^−1^ (0.95 level of confidence). *k*_1_ and *k*_2_ are the pseudo first-order and the pseudo second-order rate constants, respectively.

It should be noted that from this model, the inner diffusion was controlled by the mass transfer rate if a plot of *q*_*t*_*vs. t*^0.5^ was linear and passed through the origin. In the case of a plot that did not pass through the origin, a film diffusion adsorption mechanism as well as a chemical reaction would be the rate-controlling steps. As observed in Fig. 11, one can see that no multi-linearity was found in these plots, which could be described through a single-step occurring for the Fe^3+^, Fe^2+^, Ca^2+^ and Zn^2+^ adsorption mechanisms. In other words, the straight lines did not pass through the origin as mentioned above, indicating that the adsorption behavior of all metal cations in the aqueous phase involved a complex process. From these results, it could be concluded that the rapid intra-particle diffusion of metal cations onto CPMn occurred only *via* one-step adsorption, probably due to interactions between the active sites and cations, the high availability of free sites on external surfaces and/or the presence of easily accessible sites.^27^

### 3.8. Adsorption thermodynamics

**Fig. 12:**
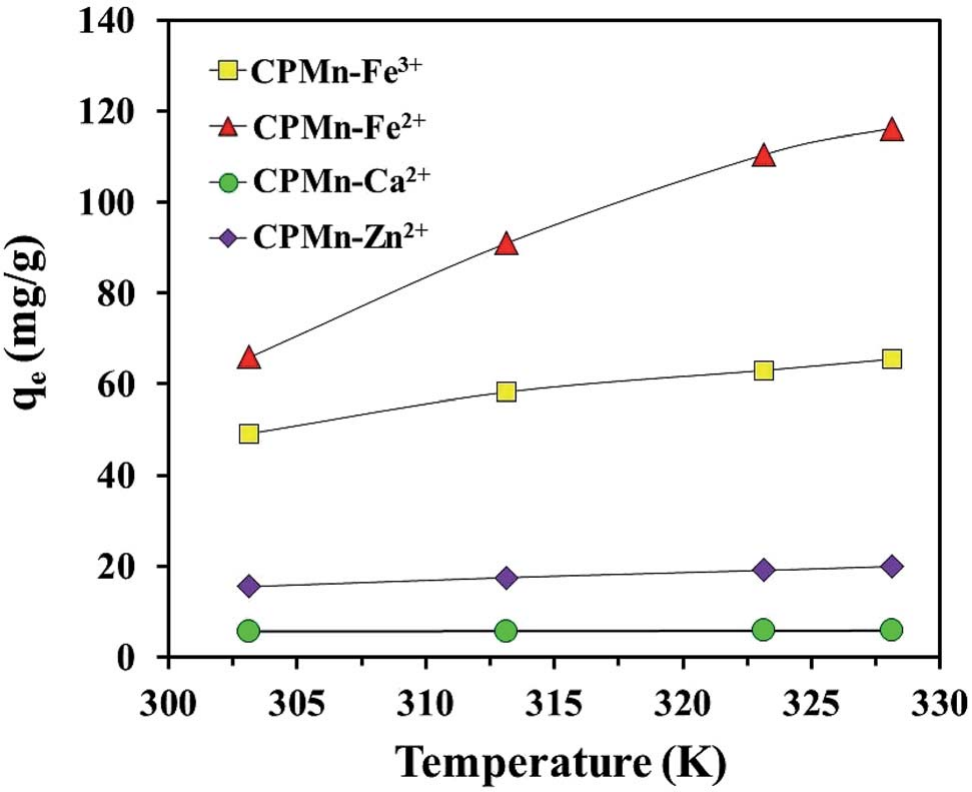
Effect of temperature on the adsorption of Fe^3+^, Fe^2+^, Ca^2+^ and Zn^2+^ by CP modified with KMnO_4_. The relative combined expanded uncertainty tSE is <±0.03 mg g^−1^ (0.95 level of confidence). The initial concentration of adsorbate (*C*_0_): Fe^3+^ = 300 mg L^−1^, Fe^2+^ = 500 mg L^−1^, Ca^2+^ = 30 mg L^−1^, Zn^2+^ = 90 mg L^−1^; 25 mL; adsorbent amount = 0.10 g; time (*t*) = 60 min; temperature (*T*) = 303.15–328.15 K; pressure (*P*) = 101 kPa.

Fig. 12 shows a comparison of the adsorption abilities for each metal cation at different temperatures using CPMn. One can obviously see that the increasing the adsorption temperature from 303.15 to 328.15 K resulted in the enhancement of the adsorption capacities for all metal cations, especially Fe^2+^ and Fe^3+^, indicating an endothermic adsorption process. This phenomenon should also be ascribed to the fact that a rise in temperature could enhance and promote the diffusion rates of metal cations across the external boundary layer and in the internal pores of the CPMn structure, as well as reduce the viscosity of the solution. To evaluate the effects of temperature on Fe^3+^, Fe^2+^, Ca^2+^ and Zn^2+^ adsorbed on CPMn, the thermodynamic parameters, namely standard enthalpy (Δ*H*, kJ mol^−1^) (Δ*H*_phys_ ≪ Δ*H*_chem_, typically Δ*H*_chem_ > 100 kJ mol^−1^), standard entropy (Δ*S*, J mol^−1^ K^−1^) and Gibbs standard free energy (Δ*G*, kJ mol^−1^), were investigated.^28^ As shown in Table 4, the negative values of Δ*G* obtained from Fe^3+^, Fe^2+^, Ca^2+^ and Zn^2+^ adsorption at different temperatures using CPMn were in a range between −0.71 and −5.36 kJ mol^−1^, suggesting a physisorption process.^29^ It should be mentioned here that the change in free energy for physisorption is in a range between −20 and 0 kJ mol^−1^, while for chemisorption it is in a range between −80 and −400 kJ mol^−1^. Here, the Δ*G* value obviously decreased to some extent with an increase in the adsorption temperature. This change in free energy could be attributed to the spontaneous natures of the Fe^3+^, Fe^2+^, Ca^2+^ and Zn^2+^ adsorption processes using CPMn. The endothermic nature of Fe^3+^, Fe^2+^, Ca^2+^ and Zn^2+^ adsorption was revealed, while positive Δ*H* and Δ*S* values indicated irreversible and randomness processes.^30,39^ A physisorption process was clearly found based on Δ*H* < 100 kJ mol^−1^, which was in good agreement with the results from the Dubinin–Radushkevich isotherm model. For comparison the adsorption performance is compared with the previous literature, as shown in Table 5, with higher adsorption capacities based on *q*_e max_ than other previously developed adsorbents. This indicates that not only was a low-cost adsorbent easily prepared, but it also presented better capacity for Fe^3+^, Fe^2+^, Ca^2+^ and Zn^2+^ adsorption. This phenomenon should be attributed to the contributions of Mn and biochar. From these results, CPMn could be considered as a promising low-cost/efficient adsorbent for the selective removal of metal cations from aqueous solution.

**Table:** The thermodynamic parameters for the adsorptions of ions onto the prepared adsorbent. The initial concentration of adsorbate (*C*_0_): Fe^3+^ = 300 mg L^−1^, Fe^2+^ = 500 mg L^−1^, Ca^2+^ = 30 mg L^−1^, Zn^2+^ = 90 mg L^−1^; 25 mL; adsorbent amount = 0.10 g; time (*t*) = 60 min; temperature (*T*) = 303.15–328.15 K; pressure (*P*) = 101 kPa.^a^ All parameters were calculated *via* nonlinear regression in the Excel program

Adsorbent	Ion type	Δ*H* (kJ mol^−1^)	Δ*S* (J mol^−1^ K^−1^)	Δ*G* (kJ mol^−1^)				*R* ^2^
				303.15 K	313.15 K	323.15 K	328.15 K	
CPMn	Fe^2+^	55.76	182.01	−0.71	−2.38	−4.32	−5.36	0.9977
	Fe^3+^	21.05	70.77	−0.88	−1.60	−2.33	−2.68	0.9999
	Ca^2+^	41.54	139.50	−1.62	−3.24	−4.46	−5.26	0.9955
	Zn^2+^	38.88	131.42	−1.91	−3.11	−4.41	−5.33	0.9930

a The relative combined expanded uncertainties tSE: standard enthalpy Δ*H* = ±0.04 kJ mol^−1^; standard entropy Δ*S* = ±0.03 J mol^−1^ K^−1^; Gibbs standard free energy Δ*G* = ±0.05 kJ mol^−1^ (0.95 level of confidence).

**Table:** A comparison of the maximum adsorption for each metal cation with other adsorbents previously reported in the literature

Adsorbent	*q*_e_ of Fe^3+^ (mg g^−1^)	*q*_e_ of Fe^2+^ (mg g^−1^)	*q*_e_ of Ca^2+^ (mg g^−1^)	*q*_e_ of Zn^2+^ (mg g^−1^)	Reference
Fe_3_O_4_@mSiO_2_–NH_2_	20.66	—	—	—	31
Hazelnut hull	13.59	—	—	—	32
Activated carbon 501	—	21.13	—	—	12
Activated biochar	—	1.46	—	—	38
Coal fly ash	—	—	21.14	—	33
Duolite C206A	—	—	64.16	—	36
Synthetic clinoptilolite	—	—	—	31.47	35
Organomontmorillonites	—	—	—	3.99	37
Reclaimed waste	—	—	—	0.081	34
CPMn	70.76	68.60	5.06	22.38	Present study

## 4. Conclusions

The preparation and modification of CP (low-cost/highly efficient adsorbent) derived from palm kernel cake was successfully achieved for Fe^3+^, Fe^2+^, Ca^2+^ and Zn^2+^ adsorption. CP was found to favor the adsorption of polar Fe^2+^ molecules, while non-polar I_2_ molecules were excellently adsorbed on ACC, as a result of the different polarities of the adsorbent surfaces. CPMn exhibited better performance for metal cation adsorption than CPHNO_3_, suggesting contributions from the carboxylic groups and MnO_2_ on the surface, which was confirmed from the TGA, FT-IR and XRD results. Fe^3+^, Fe^2+^, Ca^2+^ and Zn^2+^ adsorbed on CPMn could be easily desorbed using 1.0 mol L^−1^ HNO_3_ solution for a regeneration process. A pH range of about 6 to 7 was appropriate for the adsorption of all metal cations. The adsorption capacities for Fe^3+^, Fe^2+^, Ca^2+^ and Zn^2+^ using CPMn were 52.39, 60.15, 4.48 and 16.27 mg g^−1^, respectively, while CP without any modification showed capacities of 19.3, 36.02, 0.05 and 6.57 mg g^−1^, respectively. For the adsorption behaviors towards various metal cations using CPMn, several models were calculated with non-linear forms, such as the Langmuir, Dubinin–Radushkevich, Redlich–Peterson and Toth isotherm models, as well as a pseudo-second-order kinetic model; these were fitted based on *R*^2^ values close to 1, suggesting monolayer physisorption with a rapid adsorption process. The single-step diffusion in this study was verified using the Weber–Morris model. Moreover, the adsorption process was found to be spontaneous and endothermic in nature, as described *via* thermodynamic investigations. This research is expected to show that CPMn could be really applied to the practical process of wastewater treatment.

## Conflicts of interest

Authors declare no conflict of interest.

## Supplementary Material

RA-009-C9RA03112J-s001

## References

[cit1] Manzoor K., Ahmad M., Ahmad S., Ikram S. (2019). Removal of Pb(ii) and Cd(ii) from wastewater using arginine cross-linked chitosan–carboxymethyl cellulose beads as green adsorbent. RSC Adv..

[cit2] Mishra T., Mahato D. K. (2016). A comparative study on enhanced arsenic(v) and arsenic(iii) removal by iron oxide and manganese oxide pillared clays from ground water. J. Environ. Chem. Eng..

[cit3] Huang Y., Wu D., Wang X., Huang W., Lawless D., Feng X. (2016). Removal of heavy metals from water using polyvinylamine by polymer-enhanced ultrafiltration and flocculation. Sep. Purif. Technol..

[cit4] Vollprecht D., Krois L. M., Sedlazeck K. P., Müller P., Mischitz R., Olbrich T., Pomberger R. (2019). Removal of critical metals from waste water by zero-valent iron. J. Cleaner Prod..

[cit5] Shi Q., Terracciano A., Zhao Y., Wei C., Christodoulatos C., Meng X. (2019). Evaluation of metal oxides and activated carbon for lead removal: kinetics, isotherms, column tests, and the role of co-existing ions. Sci. Total Environ..

[cit6] Maneechakr P., Chaturatphattha P., Karnjanakom S. (2018). Adsorption behavior of As(v) from aqueous solution by using Fe^3+^-MnO_4_^−^-modified activated carbon (*Leucaena leucocephala* (Lam.) de Wit). Res. Chem. Intermed..

[cit7] Cui J., Jing C., Che D., Zhang J., Duan S. (2015). Groundwater arsenic removal by coagulation using ferric(iii) sulfate and polyferric sulfate: a comparative and mechanistic study. J. Environ. Sci..

[cit8] Zewail T. M., Yousef N. S. (2015). Kinetic study of heavy metal ions removal by ion exchange in batch conical air spouted bed. Alexandria Eng. J..

[cit9] Deng Y., Ok Y. S., Mohan D., Pittman Jr C. U., Dou X. (2019). Carbamazepine removal from water by carbon dot-modified magnetic carbon nanotubes. Environ. Res..

[cit10] Choi H. D., Jung W. S., Cho J. M., Ryu B. G., Yang J. S., Baek K. (2009). Adsorption of Cr(vi) onto cationic surfactant-modified activated carbon. J. Hazard. Mater..

[cit11] Liu W., Zhang J., Zhang C., Wang Y., Li Y. (2010). Adsorptive removal of Cr(vi) by Fe-modified activated carbon prepared from *Trapa natans* husk. Chem. Eng. J..

[cit12] Maneechakr P., Karnjanakom S. (2017). Adsorption behaviour of Fe(ii) and Cr(vi) on activated carbon: surface chemistry, isotherm, kinetic and thermodynamic studies. J. Chem. Thermodyn..

[cit13] Luo C., Tian Z., Yang B., Zhang L., Yan S. (2013). Manganese dioxide/iron oxide/acid oxidized multi-walled carbon nanotube magnetic nanocomposite for enhanced hexavalent chromium removal. Chem. Eng. J..

[cit14] Feng N. C., Fan W., Zhu M. L., Guo X. Y. (2018). Adsorption of Cd^2+^ in aqueous solutions using KMnO_4_-modified activated carbon derived from *Astragalus* residue. Trans. Nonferrous Met. Soc. China.

[cit15] Sepehr M. N., Amrane A., Karimaian K. A., Zarrabi M., Ghaffari H. R. (2014). Potential of waste pumice and surface modified pumice for hexavalent chromium removal: characterization, equilibrium, thermodynamic and kinetic study. J. Taiwan Inst. Chem. Eng..

[cit16] Bazan-Wozniak A., Nowicki P., Pietrzak R. (2018). Production of new activated bio-carbons by chemical activation of residue left after supercritical extraction of hops. Environ. Res..

[cit17] Marzbali M. H., Esmaieli M., Abolghasemi H., Marzbali M. H. (2016). Tetracycline adsorption by H_3_PO_4_-activated carbon produced from apricot nut shells: a batch
study. Process Saf. Environ. Prot..

[cit18] Rios R. R. V. A., Alves D. E., Dalmázio I., Fernando S., Bento V., Donnici C. L., Lago R. M. (2013). Tailoring activated carbon by surface chemical modification with O, S, and N containing molecules. Mater. Res..

[cit19] Ho Y. S. (2016). Isotherms for the sorption of lead onto peat: comparison of linear and non-linear methods. Pol. J. Environ. Stud..

[cit20] Huang L., Kong J., Wang W., Zhang C., Niu S., Gao B. (2012). Study on Fe(iii) and Mn(ii) modified activated carbons derived from *Zizania latifolia* to removal basic fuchsin. Desalination.

[cit21] Chang J., Ma J., Ma Q., Zhang D., Qiao N., Hu M., Ma H. (2016). Adsorption of methylene blue onto Fe_3_O_4_/activated montmorillonite nanocomposite. Appl. Clay Sci..

[cit22] Ghaedi M., Ansari A., Bahari F., Ghaedi A. M., Vafaei A. (2015). A hybrid artificial neural network and particle swarm optimization for prediction of removal of hazardous dye brilliant green from aqueous solution using zinc sulfide nanoparticle loaded on activated carbon. Spectrochim. Acta, Part A.

[cit23] Dashamiri S., Ghaedi M., Dashtian K., Rahimi M. R., Goudarzi A., Jannesar R. (2016). Ultrasonic enhancement of the simultaneous removal of quaternary toxic organic dyes by CuO nanoparticles loaded on activated carbon: central composite design, kinetic and isotherm study. Ultrason. Sonochem..

[cit24] Roy P., Mondal N. K., Das K. (2014). Modeling of the adsorptive removal of arsenic: a statistical approach. J. Environ. Chem. Eng..

[cit25] Ho Y. S., McKay G. (1999). Pseudo-second order model for sorption processes. Process Biochem..

[cit26] Vitela-Rodriguez A. V., Rangel-Mendez J. R. (2013). Arsenic removal by modified activated carbons with iron hydro(oxide) nanoparticles. J. Environ. Manage..

[cit27] Li N., Mei Z., Wei X. (2012). Study on sorption of chlorophenols from aqueous solutions by an insoluble copolymer containing β-cyclodextrin and polyamidoamine units. Chem. Eng. J..

[cit28] Hsu C. J., Chiou H. J., Chen Y. H., Lin K. S., Rood M. J., His H. C. (2019). Mercury adsorption and re-emission inhibition from actual WFGD wastewater using sulfur-containing activated carbon. Environ. Res..

[cit29] Karmacharya M. S., Gupta V. K., Tyagi I., Agarwal S., Jha V. K. (2016). Removal of As(iii) and As(v) using rubber tire derived activated carbon modified with alumina composite. J. Mol. Liq..

[cit30] Karnjanakom S., Maneechakr P. (2019). Adsorption behaviors and capacities of Cr(vi) onto environmentally activated carbon modified by cationic (HDTMA and DDAB) surfactants. J. Mol. Struct..

[cit31] Meng C., Zhikun W., Qiang L., Chunling L., Shuangqing S., Songqing H. (2018). Preparation of amino-functionalized Fe_3_O_4_@mSiO_2_ core-shell magnetic nanoparticles and their application for aqueous Fe^3+^ removal. J. Hazard. Mater..

[cit32] Sheibani A., Shishehbor M., Alaei H. (2012). Removal of Fe(iii) ions from aqueous solution by hazelnut hull as an adsorbent. Int. J. Ind. Chem..

[cit33] Kankrej S. R., Kulkarni M. S., Borhade A. V. (2018). Adsorption isotherms, thermodynamics, kinetics and mechanism for the removal of Ca^2+^, Mg^2+^ and Cu^2+^ ions onto Nosean prepared by using Coal Fly Ash. J. Environ. Chem. Eng..

[cit34] Jo Y. H., Do S. H., Kong S. H. (2014). Feasibility test for waste-reclaimed material to remove Cu^2+^ and Zn^2+^: kinetics and applications to treat a real plating wastewater. J. Environ. Chem. Eng..

[cit35] Li Y., Bai P., Yan Y., Yan W., Shi W., Xu R. (2019). Removal of Zn^2+^, Pb^2+^, Cd^2+^, and Cu^2+^ from aqueous solution by synthetic clinoptilolite. Microporous Mesoporous Mater..

[cit36] Mountadar S., Hayani A., Tahiri S., Rich A., Siniti M. (2018). Equilibrium, kinetic, and thermodynamic studies of the Ca^2+^ and Mg^2+^ ions removal from water by Duolite C206A. Solvent Extr. Ion Exch..

[cit37] Wang G., Zhang S., Hua Y., Su X., Ma S., Wang J., Komarneni S. (2017). Research paper: phenol and/or Zn^2+^ adsorption by single- or dual-cation organomontmorillonites. Appl. Clay Sci..

[cit38] Banerjee S., Mandal T., Halder G., Laminka-Ot A., Joshi S. R. (2017). Optimization of Fe^2+^ removal from coal mine wastewater using activated biochar of *Colocasia esculenta*. Water Environ. Res..

[cit39] Maneechakr P., Karnjanakom S. (2019). The essential role of Fe(iii) ion removal over efficient/low-cost activated carbon: surface chemistry and adsorption behaviour. Res. Chem. Intermed..

