# Supplementary material for: Environmental surface chemistries and adsorption behaviors of metal cations (Fe3+, Fe2+, Ca2+ and Zn2+) on manganese dioxide-modified green biochar
Source: RSC Adv. 2019 Aug 2;9(42):24074–86. doi: 10.1039/c9ra03112j (PMC9069501; doi:10.1039/c9ra03112j)
Supplement: RA-009-C9RA03112J-s001 [file RA-009-C9RA03112J-s001.pdf]

Electronic Supplementary Material (ESI) for RSC Advances.
This journal is © The Royal Society of Chemistry 2019

Supporting Information

Environmentally surface chemistries and adsorption behaviors of metal cations (Fe^{3+}, Fe^{2+}, Ca^{2+} and Zn^{2+}) onto manganese dioxide-modified green biochar

Panya Maneechakr* and Surachai Karnjanakom

Department of Chemistry, Faculty of Science, Rangsit University, Pathumthani 12000, Thailand

*Corresponding author.

E-mail addresses: panya.m@rsu.ac.th (P. Maneechakr)

1. Experimental

1.1. Adsorbent Characterization

1.1.2. Surface area, morphology, element and crystallinity

The surface area of prepared adsorbent was measured by using a Surface Area Analyzer 3Flex Micrometitics USA at liquid N_2 temperature of -196 °C using Brunauer-Emmett-Teller (BET) method. Before sorption analysis, all samples were degassed at 150 °C for 4 h. The adsorbent morphology and the existence of absorbed metal species such as Mn, Fe, Ca and Zn on adsorbent were observed using a scanning electron microscope (SEM, JSM-5410 LV) equipped with an energy dispersive spectroscopy (EDS). The formation of MnO_2 on adsorbent structure occurred from reduction of $KMnO_4$ was detected using a X-ray diffractometer (XRD, Rigaku TTRAX III Target type: Cu tube voltage = 50 kV and current = 300 mA).

1.1.3. Surface functional group, acidity-basicity and thermal decomposition

The existence of each functional group on the surface of adsorbent was verified by Fourier transform infrared spectrometry (FT-IR) using a PerkinElmer Spectrum 100 FT-IR spectrometer in the range of wavenumber between 4000 and 500 cm^{-1}. The amounts of acidic and basic functional groups on adsorbent were determined by Boehm titration method (Bazan-Wozniak et al., 2018). In brief, to quantify the amount of carboxylic, lactonic and phenolic groups, 0.5 g of adsorbent was added into 25 mL of 0.1 mol/L NaOH, 0.1 mol/L Na_2CO_3 and 0.1 mol/L $NaHCO_3$ solutions. Then, it was stirred at a speed of 150 rpm with a temperature of 30 °C for 24 h, and titrated with 0.1 mol/L HCl solution. For basicity test, the adsorbent was added into 25 mL of 0.1 mol/L HCl solution and then titrated with 0.1 mol/L NaOH solution. The thermal decomposition of carboxylic group on adsorbent was investigated using Thermogravimetric

analyzer (TGA, Mettler Toledo TGA2) at temperature range of 100-700 °C with a heating rate of 10 °C/min under N_2 atmosphere.

1.1.4. Surface charge (pH_{pzc})

A total of 0.1 g of adsorbent was added into 25 mL of 0.1 mol·L^{-1} NaCl and then pH was adjusted to remain within the range between 1 to 9. The mixture solution was stirred at temperature of 303.15 K for 30 min with a speed of 150 rpm. After complete stirring, the mixture solution was measured for surface charges by a pH Eutech instrument, pH 700 with the uncertainty of ±0.1.

1.2. Adsorption equilibrium

Here, Langmuir model was applied to point out the assumption of monolayer adsorption onto surface containing a finite number of adsorption sites without interaction between adsorbed molecules on neighboring sites and transmigration of adsorbate in the plane of surface (Huang et al., 2012) [20]. Langmuir isotherm model is given by the following Eq. (2):

$$q_e = \frac{q_{max} K C_e}{1 + K C_e} \quad (2)$$

where C_e is the equilibrium concentration of the adsorbate (mg/L), q_e is the amount of adsorption at equilibrium (mg/g), q_{max} is the Langmuir maximum adsorption capacity of adsorbate per unit mass of adsorbent (mg/g) and K is the Langmuir constant related to the adsorption equilibrium (L/mg).

For Freundlich model, it was applied to point out the assumption of multilayer adsorption taking place over a heterogeneous surface with non-uniform distribution of adsorption heat (Chang et al., 2016) [21]. The Freundlich isotherm model is given by the following Eq. (3):

$$q_e = K_F C_e^{1/n} \quad (3)$$

where K_F is the Freundlich constant related to the adsorption capacity ($mg/g(l/mg)^{1/n}$) and n is the intensity of adsorption and constants incorporating the factors affecting the adsorption capacity.

For Temkin isotherm model, it was applied until heats from the adsorption and the adsorbent–adsorbate interactions were estimated (Ghaedi et al., 2015) [22]. The Temkin isotherm model is given by the following Eq. (4):

$$q_e = (\frac{RT}{b}) \ln(AC_e) \quad (4)$$

where A is this equilibrium binding constants related to maximum binding energy (L/mol), R is the universal gas constant (8.314 $J/molK^{-1}$), T is the absolute temperature (K) and b is the Temkin constant related to heat of the adsorption.

For Dubinin-Radushkevich model, it was used to determine the porosity apparent free energy and the adsorption behavior (Dashamiri et al., 2016) [23]. The Dubinin-Radushkevich isotherm model is given by the following Eq. (5):

$$q_e = q_s \exp(-B\varepsilon^2) \quad (5)$$

where q_s is the theoretical saturation capacity (mg/g), B is the Dubinin-Radushkevich constant related to biosorption energy (mol^2/kJ) and ε is Polanyi potential which can be calculated by the following Eq. (6):

$$\varepsilon = RT \ln(1 + \frac{1}{C_e}) \quad (6)$$

Meanwhile, the free energy of adsorption (*E*, kJ/mol) was also calculated by the following Eq. (7):

$$E = \frac{1}{\sqrt{2B}} \tag{7}$$

Here, the magnitude of E might useful for estimating the mechanism of the adsorption reaction. In case of $E < 8$ kJ/mol, the physical force may affect the adsorption. Also, the adsorption was governed by ion exchange mechanism when E was in the range of 8-16 kJ/mol.

Redlich-Peterson model was applied to confirm and support the isotherm. The Dubinin-Radushkevich isotherm model is given by the following Eq. (8):

$$q_e = \frac{AC_e}{1 + BC_e^g} \tag{8}$$

Where A, B and g is the constant value by $0 < g < 1$. (g =1, Langmuir isotherm)

Toth model was proposed to apply for heterogeneous adsorption proved from potential theory by assuming to Quasi-Gaussian energy, which surface of adsorption energy had lower than maximum energy value of adsorption. The Toth isotherm model is given by the following Eq. (9):

$$q_e = \frac{q_e^{\infty} C_e}{[K_{Th} + C_e^{Th}]^{1/Th}} \tag{9}$$

where q_e^{∞} is maximum adsorption capacity (monolayer) of adsorbate per unit mass of adsorbent (mg/g), K_{Th} is the Toth constant.

1.3. Adsorption kinetic

The adsorption kinetic was simulated with pseudo first-order and pseudo second-order kinetic equations which were calculated by nonlinear regression in excel and expressed by the following Eqs. (10) and (11), respectively (Ho et al., 1999) [25]:

$$q_t = q_e(1 - e^{-k_1 t}) \qquad (10)$$

$$q_t = \frac{q_e^2 k_2 t}{1 + q_e k_2 t} \qquad (11)$$

where q_e and q_t (mg/g) are the amount of adsorbate at equilibrium and at any time, respectively. k_1 and k_2 are the pseudo first-order and the pseudo second-order rate constants, respectively. t is the contact time (min).

1.4. Adsorption thermodynamic

In this study, the adsorption process was determined at different temperatures using the following equations [24]:

$$K_d = \frac{C_0 - C_e}{C_e} \qquad (12)$$

$$\Delta G = -RT \ln K_d \qquad (13)$$

$$\Delta G = \Delta H - T\Delta S \qquad (14)$$

$$\ln K_d = \frac{\Delta S}{R} - \frac{\Delta H}{RT} \qquad (15)$$

where C_e is the equilibrium concentration of the adsorbate (mg/L), C_0 is the initial concentration of adsorbate (mg/L), R is the universal gas constant (8.314 J/mol.K), T is the absolute temperature (K) and K_d is the is distribution coefficient.

References

[20] Huang, L., Kong, J., Wang, W., Zhang, C., Niu, S., Gao, B., 2012. Study on Fe(III) and Mn(II) modified activated carbons derived from *Zizania latifolia* to removal basic fuchsin. Desalination 286, 268-276.

[21] Chang, J., Ma, J., Ma, Q., Zhang, D., Qiao, N., Hu, M., Ma, H., 2016. Adsorption of methylene blue onto Fe_3O_4/activated montmorillonite nanocomposite. Appl. Clay Sci. 119, 132-140.

[22] Ghaedi, M., Ansari, A., Bahari, F., Ghaedi, A.M., Vafaei, A., 2015. A hybrid artificial neural network and particle swarm optimization for prediction of removal of hazardous dye brilliant green from aqueous solution using zinc sulfide nanoparticle loaded on activated carbon. Spectrochim. Acta, Part A 137, 1004-1015.

[23] Dashamiri, S., Ghaedi, M., Dashtian, K., Rahimi, M.R., Goudarzi, A., Jannesar, R., 2016. Ultrasonic enhancement of the simultaneous removal of quaternary toxic organic dyes by CuO nanoparticles loaded on activated carbon: Central composite design, kinetic and isotherm study. Ultrason. Sonochem. 31, 546–557.

[24] Roy, P., Mondal, N.K., Das, K., 2014. Modeling of the adsorptive removal of arsenic: A statistical approach. J. Environ. Chem. Eng. 2, 585-597.

[25] Ho, Y.S., McKay, G., 1999. Pseudo-second order model for sorption processes. Process Biochem. 34, 451–465.

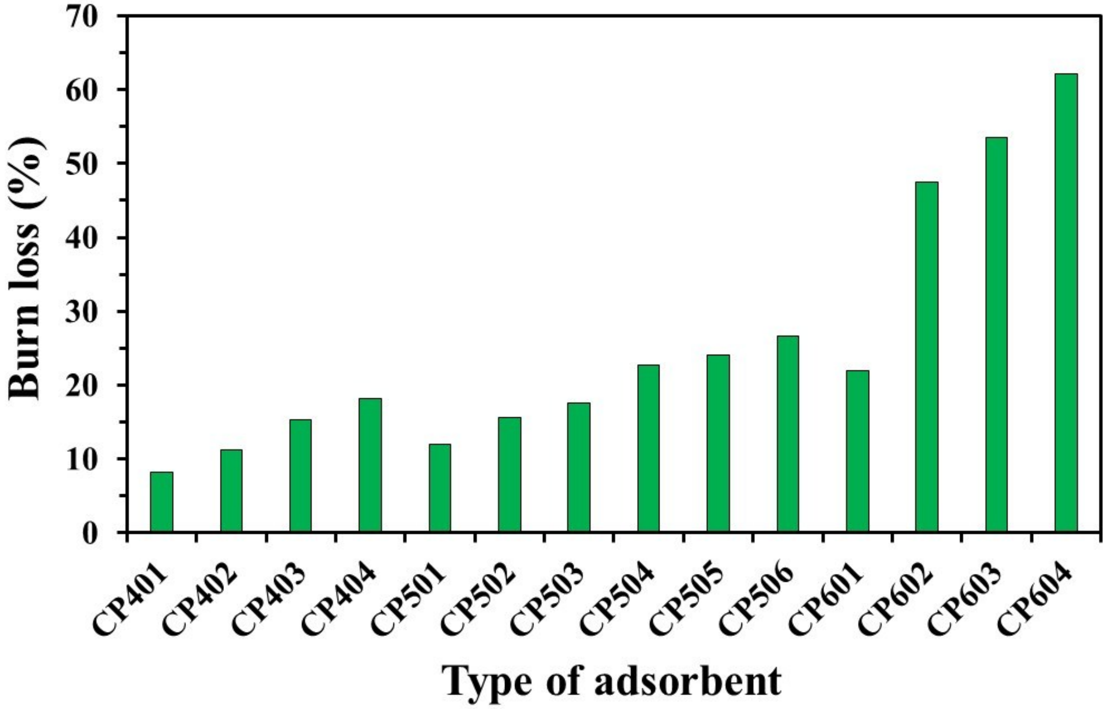

Fig. S1. Burn loss of carbonized carbons at temperature of 400-600 °C for 1-6 h.

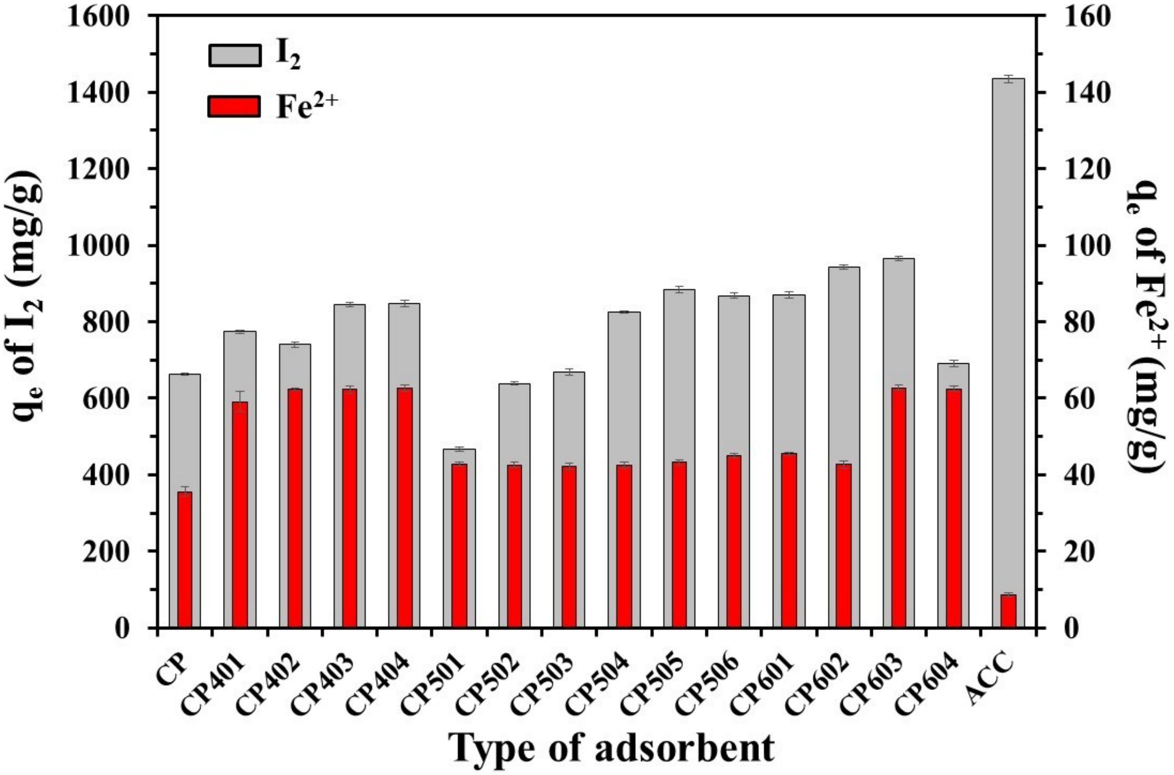

Fig. S2. Comparison of I_2 and Fe^{2+} adsorption efficiencies using various adsorbents before and after physical activation.

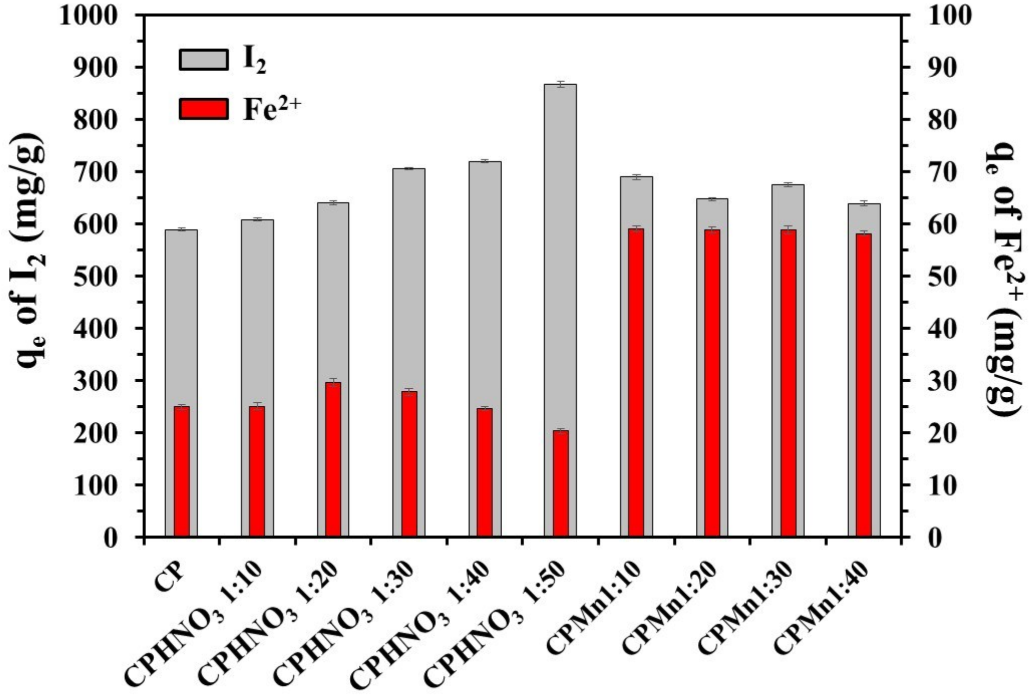

Fig. S3. Comparison of I_2 and Fe^{2+} adsorption efficiencies using various adsorbents before and after modification at different ratio of CP to $KMnO_4$ or HNO_3.

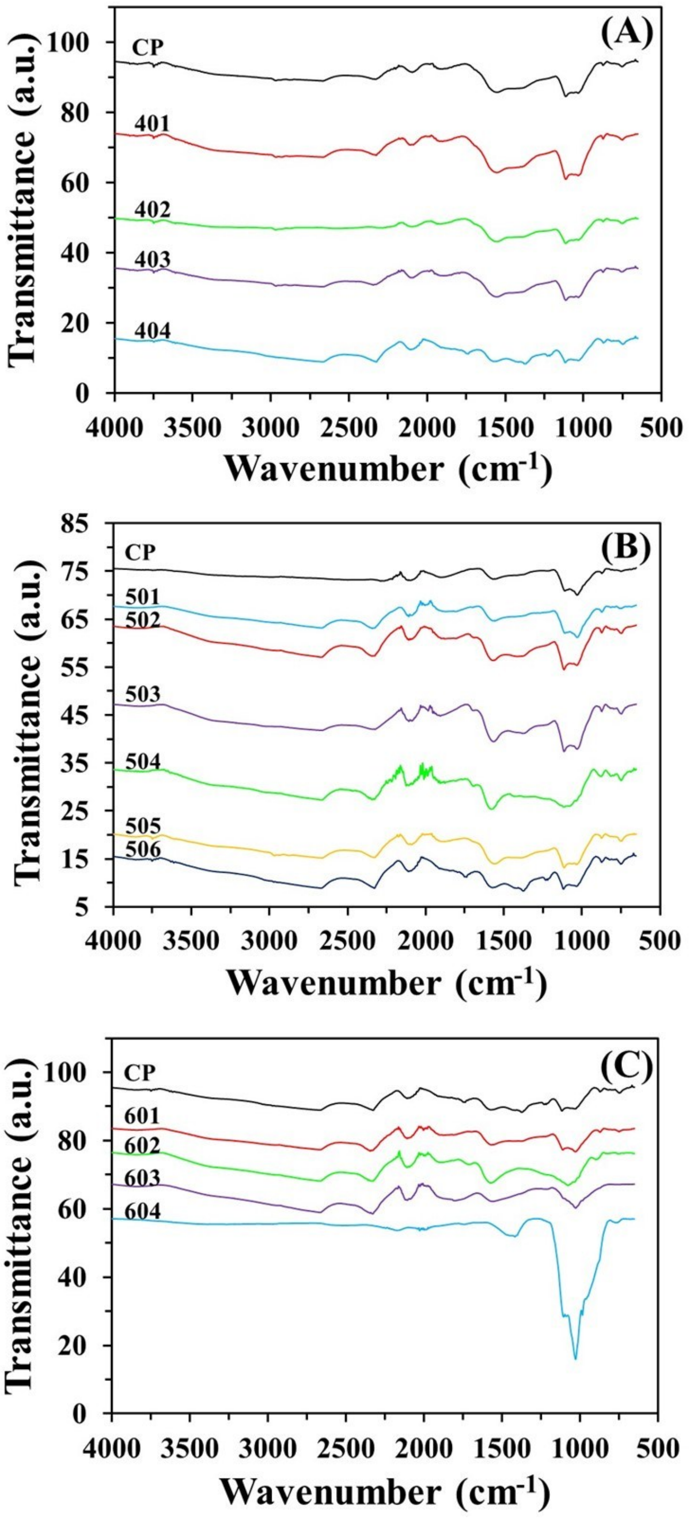

Fig. S4. FT-IR spectra of (A) CP401-404, (B) CP501-506 and (C) CP601-604.

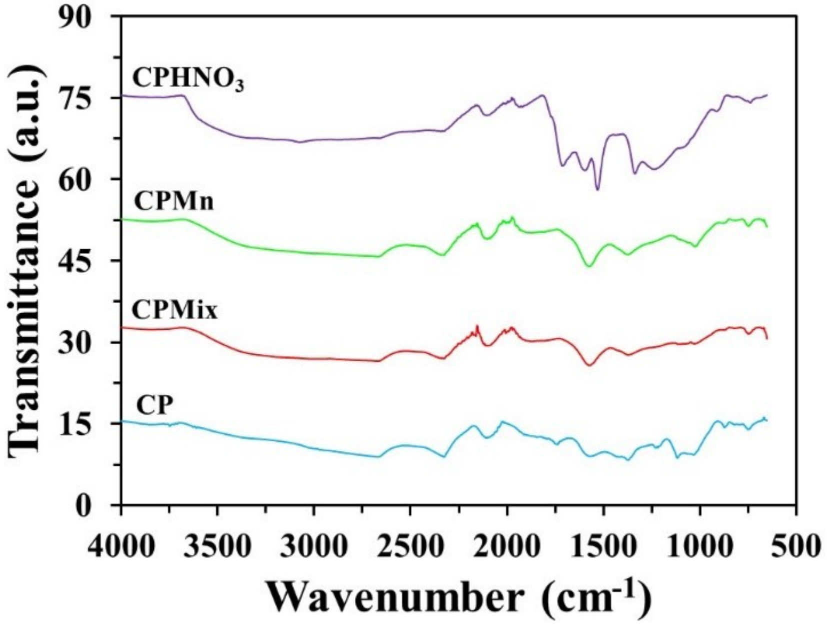

Fig. S5. FT-IR spectra of CP before and after modification with $KMnO_4$ and HNO_3.

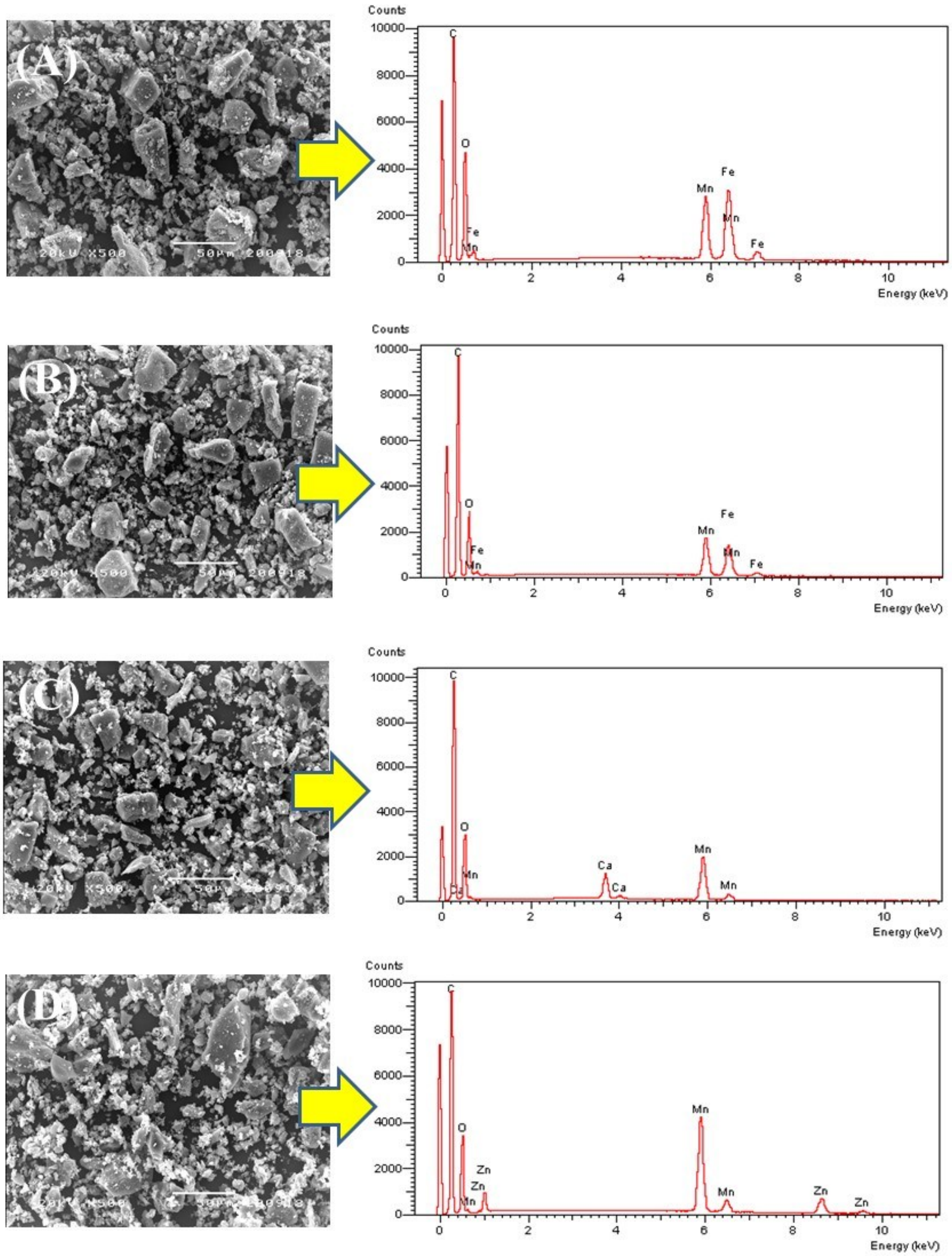

Fig. S6. SEM-EDS of CPMn after adsorption: (A) CPMn-Fe^{3+}, (B) CPMn-Fe^{2+}, (C) CPMn-Ca^{2+} and (D) CPMn-Zn^{2+}.
